# Genetic Alterations in Renal Cancers: Identification of The Mechanisms Underlying Cancer Initiation and Progression and of Therapeutic Targets

**DOI:** 10.3390/medicines7080044

**Published:** 2020-07-29

**Authors:** Ugo Testa, Elvira Pelosi, Germana Castelli

**Affiliations:** Department of Oncology, Istituto Superiore di Sanità, Vaile Regina Elena 299, 00161 Rome, Italy; elvira.pelosi@iss.it (E.P.); germana.castelli@iss.it (G.C.)

**Keywords:** renal cell cancer, genomic landscape, targeted therapy, tumor evolution, tumor heterogeneity

## Abstract

Renal cell cancer (RCC) involves three most recurrent sporadic types: clear-cell RCC (70–75%, CCRCC), papillary RCCC (10–15%, PRCC), and chromophobe RCC (5%, CHRCC). Hereditary cases account for about 5% of all cases of RCC and are caused by germline pathogenic variants. Herein, we review how a better understanding of the molecular biology of RCCs has driven the inception of new diagnostic and therapeutic approaches. Genomic research has identified relevant genetic alterations associated with each RCC subtype. Molecular studies have clearly shown that CCRCC is universally initiated by Von Hippel Lindau (VHL) gene dysregulation, followed by different types of additional genetic events involving epigenetic regulatory genes, dictating disease progression, aggressiveness, and differential response to treatments. The understanding of the molecular mechanisms that underlie the development and progression of RCC has considerably expanded treatment options; genomic data might guide treatment options by enabling patients to be matched with therapeutics that specifically target the genetic alterations present in their tumors. These new targeted treatments have led to a moderate improvement of the survival of metastatic RCC patients. Ongoing studies based on the combination of immunotherapeutic agents (immune check inhibitors) with VEGF inhibitors are expected to further improve the survival of these patients.

## 1. Introduction

Renal cell cancer (RCC) accounts for about 4% of all adult tumors. In the United States there are approximately 74,000 new cases, 5% of all tumors in male and 3% in female and almost 15,000 deaths from RCCs each year, 3.2% of all cancer deaths in male and 1.7% of all cancer deaths in female [1]. It was estimated a probability of 2.2% in male and 1.2% in female of developing a kidney cancer from birth to death [1]. In the European Community, RCC accounts for approximately 84,000 cases each year and 35,000 deaths, as estimated in 2002 [2]. RCC incidence and mortality were evaluated in the United States during the last two decades showing that: The incidence of this tumor type initially increased by 2.4% per year but later became stable since 2008; the incidence of clear cell subtype continued to increase; RCC-related mortality declined since 2001, but mortality of RCC with metastases starts to decrease only from 2012 [3]. Men are more affected than women. The highest incidence rates of RCC were observed in Eastern Europe and North America, while its mortality rates were highest in European countries [4]. Kidney cancer incidence was positively correlated with human development index and gross domestic product [4].

RCC is a complex disease entity with different histological subtypes, characterized by distinct clinical and pathophysiological features; three main histological subtypes have been identified: clear cell RCC (CCRCC), papillary RCC (PRCC), and chromophobe RCC (CHRCC). In addition, there are some less frequent subtypes, such as transitional cell carcinoma, nephroblastoma or Wilms’ tumor, collecting duct RCC, medullary RCC, tubulocystic RCC oncocytoma, and RCC associated with MiTF family translocation [5]. The most common type of RCC is CCRCC, responsible for about 75% of cases; PRCC is subdivided according to histological criteria into type I PRCC (basophilic) and type II PRCC (eosinophilic) accounting for about 15% of all RCCs; CHRCC, accounting for about 5% of RCC cases.

Hereditary cases are responsible for about 5% of all cases of RCC [5]. Many autosomal dominant hereditary RCC syndromes have been reported and included those in which germline pathogenic mutations at the level of *VHL, MET, FH, SDH A/B/C/D, FLCN, TSC1/TSC2, BAP1, CDC73,* and *MiTF* are involved [6]. FH and BAP1 germline RCCs are associated with more aggressive disease [6]. Familiar RCCs occurred earlier as age of onset (mean ages 37–39 years) compared to sporadic RCCs (63–64 years) [7]. Inherited RCC syndromes are thought to account for 5% of all cases [7].

Genomic research has identified relevant alterations associated with each RCC subtype, as it will be discussed below.

## 2. Hereditary RCCs

The prevalence of germline mutations in known predisposition genes and other genes associated with cancer development was explored in 254 patients with advanced RCC; about 16% carried pathogenic or seemingly pathogenic germline variants at the level of 17 different cancer-predisposition genes: 5.5% of these patients carried mutations at the level of RCC-associated genes, such as *FH, BAP1, VHL, MET, SDHA,* and *SDHB*; 10.5% of these patients carried mutations in genes not clearly associated with RCC, including the *CHEK2* gene [8]. For the genes not traditionally associated with RCC, only the *CHEK2* gene was mutated more frequently among RCC patients, compared to the general population [7]. A typical disease-associated feature of germline-associated RCCs was the early onset and multifocal disease at diagnosis [7]. The main features of hereditary RCCs are reported in Table 1.

## 3. Von Hippel-Lindau Disease

VHL disease is an autosomal dominantly inherited familial neoplastic condition with an incidence of approximately 1/30,000–1/36,000 live births and is caused by constitutional mutations at the level of the *VHL* tumor suppressor gene [8]. Germline *VHL* gene mutations predispose affected subjects to the development of benign and malignant tumors located at the central nervous system and visceral organs. Typical clinical characteristics are represented by hemangioblastomas of the brain, spinal cord, and retina; renal cysts and renal cell carcinoma; pheochromocytoma, pancreatic cysts, and neuroendocrine tumors; endolymphatic sac tumors; and ependymal and broad ligament cysts. Particularly, according to genotype-phenotype correlations VHL disease is classified into two subtypes, type 1 and 2 distinguished on the basis of the presence or not of pheochromocytoma: type 1 is associated with a very low risk of pheochromocytoma; while type 2 is associated with high risk of pheochromocytoma and is subdivided into type 2A (low risk of RCC), 2B (high risk of RCC), and 2C (only pheochromocytomas) [9]. The genotype correlates with the type of tumor risk observed in VHL syndrome: truncating or missense mutations are associated with type 1 and missense mutations with type 2 [10]. Recent studies have explored the relationship between genotype and phenotype in VHL syndrome: G239T mutation was linked with VHL type 2B, associated with renal cell carcinoma, pheochromocytoma, and cerebellar hemangioma; A232T mutation was related to VHL type I, associated with renal cell carcinoma alone; G500A mutation was associated with VHL type II, characterized by pheochromocytoma and cerebellar, retina and spinal cord hemangioblastoma; A293G mutation was associated with pheochromocytoma and thus with type IIC VHL [11]. The role of different types of germline *VHL* mutations classified as missense or truncating mutations and two subgroups of missense mutations subdivided according to mutations affecting the HIF-α binding site (HM) and mutations not affecting the HIF-α binding site (nHM) was also investigated [12]. In fact, the β-domain of the VHL protein comprises several β-sheets and binds HIF-α through residues 65–117. The results of this study showed that: (i) Missense mutations are associated with an increased risk of pheochromocytoma, but a lower risk of renal cancer than truncating mutations; among missense mutations, HM mutations conferred a higher risk than nHM mutations of developing renal cancer [12]. At prognostic level, nHM mutations were associated with a better overall survival than HM and truncating mutations [12].

In individuals with VHL syndrome the lifetime risk of developing CCRCC is over 70%, with an average age of 40–45 years, about two decades earlier than the age of presentation of sporadic RCC [13]. Patients with VHL disease and asymptomatic family members carriers of the VHL mutation are annually screened for asymptomatic tumors and starting from the age of 16 years are controlled for RCC by magnetic resonance imaging, thus these patients undergo RCC removal when the tumor mass reaches 3 cm of diameter [13].

An increased level of genetic homogeneity was observed among clear-cell RCC (CCRCCs) with germline *VHL* mutations, compared to sporadic CCRCCs; this greater homogeneity reflects the smaller number of copy number alterations occurring in VHL syndrome-associated CCRCCs [14]. Both in CCRCC with germline *VHL* mutation and in sporadic CCRCC, the most relevant copy number alterations occurred at the level of 3p deletion involving the *VHL* gene, p9 deletion involving *CDKN2A* and *CDKN2B* genes, and of 8q amplification involving the *MYC* gene amplification [14]. 

At macroscopic level, kidneys display multiple specific and solid lesions, the majority being of low-grade. Several studies have explored the evolution at clonal level of RCCs developing in individuals with germline *VHL* mutations. The genomic analysis on multi-focal RCCs developing in an individual with germline *VHL* mutation showed that tumors arising in this multifocal context are clonally independent and harbor distinct secondary events, such as loss of chromosome 3p; despite this heterogeneity, the genetic alterations converge upon PI3K-AKT-mTOR signaling pathway; the tumors display only a minimal intratumoral heterogeneity [15]. These observations suggested the development of RCC from germline *VHL* mutation, follow the evolutionary principles of complementary contingency and convergence [15]. The analysis of 40 different RCC tumor foci derived from six patients with VHL syndrome confirmed that tumor foci are clonally independent [16]. The pattern of nucleotide substitution and the number and type of copy number alterations follow an individual pattern, thus suggesting that the genetic background and the environment plays a significant role in the types of secondary genetic alterations occurring during the development of RCCs with germline *VHL* mutations [16].

Studies based on the analysis of early renal cancers derived from nephrectomies performed in VHL disease patients provided evidence that biallelic inactivation of *VHL* is observed in preneoplastic renal lesions, in association with HIF activation [17]. It is important to note that in Von Hippel-Lindau disease only one of the two *VHL* alleles carries a germline mutation; therefore, in these patients, the inactivation of the second allele is one of the first events during renal cancer development. Biallelic *VHL* inactivation is also required for the development of sporadic renal cancer, but requires a longer time than in VHL disease since the two *VHL* alleles must be inactivated [18]. Sporadic CCRCC displays loss of the short arm of chromosome 3 (observed in ≥90% of patients), with a deletion region encompassing four tumor suppressor genes that are also frequent targets for inactivating point mutations on the other chromosomal allele: *VHL* (with point mutations in 60–70% of cases and epigenetic silencing in about 5–10% of patients), *PBRM1* (40%), *BAP1* (10%), and *SETD2* (10%). In CCRCC developing in patients with VHL disease, one allele is mutated via germline mutation, whereas the other allele is lost by 3p chromosome loss. Both in sporadic and VHL-hereditary CCRCC the most common cause of 3p loss is a chromotripsis event leading also to concurrent 5q chromosome gain [18]. In VHL disease, one allele is altered through germline mutations and this explains the high penetrance and the accelerated RCC development observed in these patients [18].

The retrospective study analysis of the natural history of RCC developing in VHL disease showed that: (i) The mean age of onset was 38.8 years, with a mean initial tumor size of 3.1 cm; (ii) the mean tumor growth rate was 0.49 cm/year; (iii) some factors, such as later age of onset, larger initial tumor size, missense mutation, mutations located at the level of exon 3, were associated with faster tumor growth; (iv) bilateral tumors, large initial tumors, fast tumor growth, and presence of metastases are high-risk factors for poor prognosis in germline VHL-related RCCs [19].

## 4. Hereditary Papillary Renal Carcinoma Type I

Hereditary papillary renal carcinoma is an autosomal dominant syndrome with a predisposition to the development of bilateral and multifocal type I papillary renal cell cancer. Germline mutations located at the level of the tyrosine kinase domain of the hepatocyte growth factor receptor, *c-Met*, are responsible for hereditary papillary renal cell cancer (HPRCC) type I, a very rare form of familial kidney cancer [20]. The mutants *c-Met* observed in these patients in suitable cellular and animal models display enhanced and dysregulated kinase activity and induce cell transformation and tumorigenicity [20]. A fundamental study by Schmidt and coworkers in 1997 led to the identification of missense mutations located in the tyrosine kinase domain of the *MET* gene in the germline of affected members of HPRCC families, as well as in a subset of sporadic PRCCs [21]. The same authors in a study on PRCCs identified 13% of cases with *c-MET* mutations: half of these patients were found to harbor germline *c-MET* and the rest only somatic *c-MET* mutations [22]. Interestingly, these patients, including those with germline c-MET mutations do not have a history of familial disease related to HPRCC [22]. MET mutations cause constitutive activation of the cytoplasmic domain of the receptor, stimulate cell growth, and represent the main pathogenetic event in the development of HPRCC. Direct DNA diagnosis in HPRCC is based on the identification of mutations at the level of *MET* exons 15–21, encoding the cytoplasmic domain of the receptor.

HPRCC is characterized by multiple, bilateral neoplasms which are hypovascular; the disease is usually indolent and diagnosed at radiological examination [23]. Papillary renal neoplasms from both patients with hereditary or somatic c-MET mutations share the same histologic features typical of chromophil basophilic type I PRCC, including macrophages and psammoma bodies; a papillary and/or tubulopapillary architecture is observed in all these tumors; clear cells were commonly detected in variable proportions in all c-*MET*-mutated PRCCs [23].

In addition to *c-MET* mutations, other genetic abnormalities are commonly observed in HPRCCs: trisomies of chromosomes 7 and 17 are common in HPRCCs [24]; trisomy 7 harboring non-random duplication of the mutant *c-MET* proto-oncogene seems to play a significant role in the development of multiple renal tumors [25,26]; multifocal bilateral renal tumors of hereditary PRCC develop as different clones in the renal parenchyma [25]. Interestingly, a case of a family with HPRCC was reported with a novel germline missense mutation of *c-MET* with a histological pattern consisting in multiple adenomas and papillary renal cell carcinomas with focal clear cells and a mixture of type I and type II pattern [27].

Interestingly, for the treatment of patients with advanced disease, the use of c-MET inhibitors was explored. Thus, foretinib, a pan-kinase inhibitor of MET, VEGFR2, RON, and AXL, was evaluated in patients with PRCC, showing 50% of partial responses among patients with HPRCC and 20% in PRCC patients with somatic *c-MET* mutations [28].

## 5. Germline PTEN Mutation Cowden Syndrome

Cowden syndrome, or PTEN hamartoma tumor syndrome, is a rare (estimated incidence of 1 in 200,000 individuals) cancer syndrome, inherited in an autosomal dominant pattern, with a penetrance up to 90% in the second decade. The majority of patients with this syndrome were found to have germline mutations in *PTEN* [29]. These patients develop multiple hamartomas and are at increased risk for breast, endometrial, thyroid, and kidney cancers. RCC in Cowden syndrome is predominantly of the papillary and chromophobe type, beginning around 40–50 years. Mester and coworkers analyzed a cohort of patients with Cowden syndrome and RCC and estimated that these patients had a >30 fold increased risk of developing renal cancer [30].

Shuch et al. reported the study of 24 patients with Cowden syndrome and observed the development of RCC in 4 of these patients: three with solitary tumors, two with papillary type I histology and one with clear cell histology; one patient with bilateral, synchronous chromophobe tumors [31].

A recent study reported an atypical presentation of Cowden syndrome in a subject with heterozygous mutation C1003T in the *PTEN* gene, who developed four primary onset carcinomas (one melanoma, two CCRCC, and a follicular variant of papillary thyroid carcinoma). Interestingly, the analysis of family’s genetic background identified deleterious variants in two candidate modifier genes: *CECAM1* and *MIB2*; *CECAM1* is a tumor suppressor gene which presents loss of expression in RCC [32]. 

## 6. Hereditary BAP1 Tumor Syndrome

The BRCA1-associated protein1 (BAP1) syndrome is a tumor predisposition syndrome dependent on the presence of germline pathogenic variants at the level of the tumor suppressor gene *BAP1* that predisposes to the development of various types of tumors including uveal melanoma, mesothelioma, cutaneous melanoma, and RCC [33]. The first null variants were described in patients with uveal melanoma [34] and melanocytic tumors [35]. Subsequent studies have reported patients developing RCCs [36,37]. However, the incidence of RCC in these patients is less frequent than that of melanoma and mesothelioma [33]. The median age of RCC development in these patients is around 50 years [33]. The three most frequently observed missense mutations in these patients are H94R, L100P, and T173C [33]. Popova et al. identified in a family prone to RCC a germline mutation of *BAP1* gene (277A>G; Thr93Ala); furthermore, these authors screened 11 families that included individuals carrying germline deleterious *BAP1* mutations and 6 of these families presented with RCC-affected individuals [36]. Farley et al. reported a family with a BAP1 germline variant (41T>A; L14H), disrupting a highly conserved residue in the catalytic domain: 22% of the individuals of this family display RCC, mostly multifocal and of the clear cell type [37].

The evaluation of the growth rates of a cohort of 292 patients with genetically defined renal tumors and showed that BAP1-deficient tumors are those growing at the fastest rates [38]. 

## 7. Succinate Dehydrogenase (SDH) and Fumarate Hydratase (FH)-Deficient Renal Cell Carcinoma

SDH, member of the Krebs cycle and electron transport chain, is a mitochondrial enzyme complex composed of four subunits (SDHA, SDHB, SDHC, and SDHD). Germline mutations of the genes encoding the SDH subunits result in hereditary syndromes associated with the development of paraganglioma-pheochromocytoma, gastrointestinal stromal tumors, and RCC [39].

FH deficiency is a rare autosomal recessive hereditary syndrome, resulting in the homozygote condition, in a severe defect of cellular metabolism, associated with progressive encephalopathy and, in the heterozygote condition, in the predisposition to develop an early-onset kidney cancer syndrome [40].

Most of the renal tumors developing in individuals with SDH deficiency, particularly those associated with germline *SDHB* mutations, exhibit a distinctive morphology consisting in tumors composed by cuboidal cells with bubbly eosinophilic cytoplasm, arranged in solid nests or in tubules surrounding central spaces [41].

Gill et al. have reported SDH-deficient renal carcinomas from 27 patients and estimated that 0.05%–0.2% of all carcinomas are SDH deficient; 94% of these tumors displayed the typical morphology of SDH-deficient renal cancers; all the patients performing a genetic evaluation displayed germline *SDHB* mutations (only in one patient *SDHA* mutations were detected); a part of these patients had a metastatic disease, associated with high-grade nuclear atypia or coagulative necrosis [42]. Williamson et al. reported the characterization of 11 SDH-deficient RCC and observed the common presence of intratumoral mast cells; the majority of patients with *SDHB* gene mutations exhibited also loss of the second allele [43]. These studies have supported the existence of a unique subtype of renal cell carcinoma, characterized by SDH deficiency [43]. In line with these studies, SDH-deficient renal cell carcinoma was accepted as a specific tumor type in the World Health Organization Classification [5].

In some rare patients, *SDH* mutations may co-occur with *Xp11* translocation RCC, characterized by *TFE3* chromosomal translocations involving break points in the *TFE3* gene; renal cell carcinomas with translocations make part of *MiT* family translocation renal cell carcinoma and are composed by eosinophilic cells, with cytoplasmic inclusions and prominent nucleoli [44].

Gupta et al. have recently reported the results of a screening of SDHA/B deficiency in a group of 1009 renal cell neoplasms: SDH-deficient renal cell cancers were detected only in the cases originally classified as oncocytomas (1.1% of these tumors) [45].

Ajamir et al. have performed a systematic review of all the main studies reporting SDH-mutant renal cell carcinoma [46]. The most commonly mutated gene was *SDHB* (with 137G>A being the most frequent mutation) and less frequently *SDHC* (380A>G being the most frequent mutation) and *SDHA* [46]. Rare cases of SDH-deficient renal cell cancers are related to alterations of the *SDHA* gene: Yakirevich et al. reported a case of SDH-deficiency RCC, characterized by homozygous deletion of the *SDHA* gene (9 of the *SDHA* gene exons were deleted): at immunohistochemical level, the expression of both *SDHA* and *SDHB* was lost [47].

The characterization of an SDHB-deficient RCC cell line isolated from young patient carrying the SDHB^R46Q^ mutation was used as a tool to elucidate the alterations of metabolism caused by SDH deficiency [48]. SDHA catalyzes the oxidation of succinate to fumarate with the reduction of FAD^+^ to FADH_2_; three iron-sulfur (Fe-S) clusters present in SDHB improve the transfer of electrons from FADH_2_ to ubiquinone, bound by SDH through the SDHC and SDHD subunits. The SDH function and molecular organization require two conserved L(I)YR motifs present in SDHB; the *SDHB^R46Q^* mutation impairs one of these two L(I)YR motifs, by changing IYR to IYQ and thus determining an incapacity of SDHB to incorporate Fe-S cluster, with its consequent unstability [49]. SDHB-deficient renal tumor cells displayed a marked change in their energetic metabolism with a shift to aerobic glycolysis and marked decrease of oxidation phosphorylation, with very low entry of glucose into TCA cycle metabolites. As a consequence of SDHB degradation, SDHB-mutant cells displayed markedly decreased oxygen consumption, increased succinate levels, and pronounced use of glutamine as the main source of TCA cycle metabolites via reductive carboxylation (reduction of glutamine-derived α-ketoglutarate into citrate) [48]. The metabolic changes determine also an increase of HIF-1α, but not HIF-2α levels, and a marked DNA cycle island methylator phenotype [48]. Through the study of SDHB-ablated kidney mouse cells it was shown that lack of SDH activity induces the commitment of the cells to consume extracellular pyruvate, inducing Warburg-like bioenergetic features; pyruvate carboxylation shifts glucose-derived carbons into aspartate biosynthesis and, through this mechanism, sustains tumor cell growth [50]. 

SDH inactivation leads to a massive accumulation of succinate, acting as an oncometabolite. Succinate levels, assessed on tumor biopsies are a high specific biomarker of SDH-mutated tumors. Succinate can be detected in vivo by magnetic resonance spectroscopy (MRS). A pulsed proton magnetic resonance spectroscopy (1)H-MRS sequence was developed, optimized, and applied to imaging of patients with paraganglioma: a succinate peak was detected at 2.44 ppm in all paraganglioma patients carrying an *SDHx* gene mutation, but not in patients exempt of *SDHx* mutation [51]. Potential suitable applications of this technique include non-invasive diagnosis and disease stratification, extended also to monitoring of tumor response to anticancer treatments [52].

Succinate accumulated in individuals with germline *SDHx* mutations acts as an oncometabolite and is responsible at a large extent for the oncogenic effect mediated by SDH mutational deficiency [53,54,55]. Thus, succinate deregulates the HIF pathway through a direct inhibition of prolyl hydroxylases (PHDs), targeting HIF for degradation [56]. The stabilization of HIF1α and HIF2α causes an upregulation of downstream HIF targets, such as VEGF and GLUT1 [57] and the consequent generation of hypoxic and highly vascularized phenotypes [58]. In addition to the induction of a pseudohypoxic phenotype, succinate exerts other biological activities involved in its protumorigenic effects: (i) epigenetic dysregulation due to direct inhibition of histone lysine demethylases (KDM) and TET2, with consequent hypermethylation phenotypes and alteration of the expression of multiple genes involved in the control of cell proliferation and differentiation [59]; (ii) activation of succinate receptor (SUCNR1) with consequent activation of angiogenic proteins; (iii) post-translational protein modification through a process of succinylation; (iv) dependency on pyruvate carboxylase to funnel pyruvate into the truncated TCA cycle for biosynthesis of aspartate [54].

A recent study discovered a potential vulnerability of hereditary SDH-deficient RCCs related to a peculiar sensitivity to synthetic-lethal targeting poly(ADP)-ribose polymerase (PARP) inhibitors [60]. This peculiar sensitivity is due to the capacity of succinate to suppress the homologous recombination (HR) DNA-repair pathway required for the reparation of DNA double-strand breaks and for maintenance of genome integrity [60].

Germline mutations in *FH* predispose to dominantly inherited uterine fibroids, skin leiomyomata, and aggressive papillary renal cancer; according to these observations, it was proposed that *FH* acts as a tumor suppressor [61]. For the frequent occurrence of cutaneous and uterine leiomyomas this hereditary syndrome is also known as hereditary leiomyomatosis and renal cell carcinoma (HLRCC). FH-deficient renal cell cancers can occur also sporadically: thus Pan et al. have investigated 13 patients with FH-deficient renal cancers and observed absent expression in 12/13 cases, germline *FH* mutations in seven cases, and somatic mutations of *FH* gene in the remaining four cases [62].

Linehan and coworkers reported a comprehensive characterization of papillary RCCs; in this context, they identified a subset of papillary type 2 RCCs, characterized by increased DNA methylation at the level of loci unmethylated in corresponding normal cells (CIMP, CpG Island Methylator Phenotype) [63]. These tumors correspond to 5.6% of all PRCCs and were characterized by: (i) Universal methylation of *CDKN2A* promoter, and germline or somatic mutations of FH (4 patients displayed germline *FH* mutations and one showed somatic *FH* mutations); low *FH* mRNA expression, associated with increased expression of genes associated with cell-cycle progression and response to hypoxia [63]. Chen and coworkers have performed an extensive molecular analysis of 62 cases of RCC with unclassified histology and observed FH deficiency in 6% of these tumors [64]. These four cases were FH-negative and 2SC-positive at immunohistochemical level and in 3/4 cases harbored germline *FH* mutations and in 1/4 somatic *FH* mutations [64]. 

Germline *FH* mutations are observed in about 90% of families with HLRCC [65]. The remaining cases, apparently negative for *FH* mutations, could lack point mutation for several different reasons, including the presence of inactivating mutations in noncoding gene regions (promoter or enhancer) or deletion of the *FH* gene. In cases positive for *FH* mutations, the most frequent mutations located along the entire length of the coding region were represented by missense and frameshifts, and more rarely, by non-sense and splice site mutations [65]. In a large series of HLRCC patients, 68 different germline mutations of the *FHG* gene were identified: 18 truncating or frameshift mutations, 37 missense mutations, 9 splice-site, and 4 large deletions [66].

Vocke et al. have explored the occurrence of *FH* gene mutations in a group of patients with phenotypic manifestations consistent with HLRCC reporting in the 13 families explored, 11 complete *FH* gene deletions, and 2 partial *FH* gene deletion; kidney cancer was diagnosed in 32% of these patients and in 54% of families possessing either complete or partial *FH* deletions [67]. These observations clearly indicate that *FH* gene deletions, as well as gene mutations are associated with the development of RCCs [67].

The histologic growth patterns of FH-deficient tumors are heterogeneous: the large majority of these cases exhibited multiple histologic growth patterns, with papillary being the most frequent histotype (52%), followed by solid (21%), cribiform/sieve-like (14%), sarcomatoid (3%), tubular (3%), cystic (3%), and low-grade oncocytic (3%) [68]. Forde et al. showed that the histopathologic features of 18 cases of FH-deficient RCCs were variable, with 7/18 CCRCC, 9/18 PRCC (6/18 type 2 PRCC), 1 collecting duct cancer, 1 with oncocytic cystic morphology [69]. Median age of RCC onset was 44 years [69]. Pan et al. reported the clinicopathologic features of 13 cases of FH-deficient RCCs and subdivided these tumors according to the features of nuclei: The presence of typical big nuclei with or without eosinophilic nucleoli (observed in 11/13 case) were associated with disease progression or death; the presence of low-grade nuclei and eosinophilic cytoplasm (observed in 11/13 cases) showed no disease progression [62]. Furuya et al. recently reported the clinicopathologial and molecular features of 13 Japanese patients with hereditary FH-deficient renal cell carcinomas: most tumors had type 2 papillary architecture or tubulocystic pattern or both; at immunohistochemical level, 10 tumors were positive for PD-L1; somatic mutation analysis showed loss of heterozygosity of *FH* in 10 tumors [70]. 

In HLRCC subjects the most frequent age of RCC development is 40–50 years. In a minority of FH-deficient patients RCC development occurs in patients aged younger than 20 years; a significant proportion of these young patients exhibited a metastatic disease [71].

FH deficiency in RCC determines a marked alteration of energetic metabolism. *FH* gene encodes for the TCA cycle gene fumarate hydratase, responsible for the bidirectional conversion of fumarate and L-malate. HLRCC-related RCCs display a marked FH deficiency in these cells, one allele is germline mutated and the other allele is somatically lost. The FH deficiency in kidney cancer cells determines a marked metabolic remodeling, with changes at the level of glucose and glutamine metabolism and of mitochondrial respiration. Particularly, FH-deficient cancer cells undergo a Warburg metabolic shift characterized by aerobic glycolysis and reduced oxidative phosphorylation [72,73,74,75]. Isotope tracer studies in FH-deficient renal cancer cells showed that the contribution of glucose-derived carbon to TCA cycle is very limited, whereas glutamine-derived carbon enters the TCA cycle through reductive carboxylation of α-ketoglutarate [72,73,74,75]. 

The glycolytic shift induced by fumarate deficiency induced several consequences at the level of the AMP-activated pathway (AMPK): (i) AMPK levels were decreased with consequent lowered expression of the iron transported DNMT1; (ii) in turn, reduced DNMT1 levels induced a condition of cytosolic iron deficiency, activating the iron regulatory proteins, IRP1 and IRP2, and increasing the expression of HIF-1α; (iii) activation of AMPK or silencing of HIF-1α decreases the invasive properties of FH-deficient renal cancer cells [76]. 

Fumarate promotes tumorigenesis through various mechanisms: (i) by reversibly inhibiting dioxygenase involved in epigenetic signaling: fumarate inhibits TET-mediated demethylation of a DNA region involved in the regulation of the antimetastatic miRNA cluster 6 *miR-200ba249*, inducing the expression of transcription factors involved in the activation of epithelial-to-mesenchymal (EMT) [77]; fumarate is a competitive inhibitor of 2-oxoglutarate-dependent prolyl hydroxylase domain: containing proteins (PHD) that hydroxylate HIF and this inhibition lead to HIF stabilization [78] by inducing post-translational protein modification through succinylation due to the peculiar capacity of fumarate to interact with specific cysteine residues [79,80].

Several potentially important targets of succination have been identified in FH-deficient renal cancer cells: (i) Fumarate induces succination of key components of the iron-sulfur cluster biogenesis family of proteins, inducing defects in the biogenesis of iron-sulfur clusters that affect the function of the complex I of respiratory chain [81]; (ii) succinate targets the protein Kelch-like ECH-associated protein-1 (KEAP1), abrogating its repressive effects on the transcription factor NRF2 and thus resulting in upregulation of NRF2-dependent genes involved in the regulation of key antioxidant pathway mediating the capacity of cells to adapt to oxidative stress [82,83]. In line with these findings, NRF2 as well as downstream NRF2 target genes are upregulated in FH-deficient renal cancers [82,83].

Interestingly, somatic mutations in *NRF2, CUL3,* and *SIRT1*, rarely observed in PRCC2, are responsible for driving the NRF2 activation phenotype in these tumors [84]. In addition to these effects on KEAP1, fumarate can react with the sulfur atom of glutathione to generate succinated glutathione, thus inhibiting the function of glutathione and resulting in increased oxidative stress in FH-deficient RCCs [85,86].

Kulkarni et al. have recently reported the results of a study based on the use of chemoproteomic probes to explore the spectrum of occupancy of fumarate-reactive cysteines and identified an FH-sensitive cysteine in SMARCC1, a member of the SWI-SNF ((Switch/Sucrose Non Fermentable) ATP-dependent chromatin remodeling complexes [87].

Interestingly, a proteasomal inhibitor, marizomib, disrupts glucose and glutamine metabolism in HLRCC cells via inhibition of glycolysis and lowered expression of glutaminases, thus restricting nutrients and the cells’ antioxidant response capacity, supporting a potential use of proteasome inhibitors in HLRCC [88].

## 8. Birt-Hogg-Dubé (BHD) Syndrome

BHD syndrome is an autosomal dominant inherited disease that predisposes at-risk individuals to develop benign cutaneous fibrofolliculomas, pulmonary cysts, spontaneous pneuomothoraces, and increased risk for renal cancer. Renal tumors that develop in the context of BHD syndrome are heterogenous and are frequently bilateral with various histologies. Through the study of numerous families inheriting the mutated gene responsible for BHD syndrome it is estimated an increased risk of developing RCC for BHD-affected family members of about 7-fold in comparison with unaffected individuals [89]. Various histologic types of RCC are associated with DHB syndrome, including hybrid oncocytic tumor (50%) with histological features of both chromophobe RCC and renal oncocytoma; chromophobe RCC (35%); CCRCC (9%); renal oncocytoma (5%) [90,91]. A peculiar histologic finding of these tumors is represented by the presence of so-called “oncocytosis” defined as a pathological condition in which renal parenchyma is diffusely involved by numerous oncocytic nodules [92]. These foci of oncocytic cells have been suggested to represent the precursor lesions of BHD-associated tumors [90,91]. A recent study performed on clinicopathologic information on 220 families with BHD syndrome confirmed the consistent histologic heterogeneity of BHD-associated kidney tumors, with 43% of the chromophobe subtype and 34% of the hybrid oncocytic/chromophobe subtype; 64% of the patients with renal cancer had multiple lesions at the time of genetic diagnosis [93]. 

In 2002, genetic linkage studies in BHD families allowed the localization of the gene responsible for BHD syndrome at the level of chromosome *17p11*, and the identification of this gene as the folliculin (*FLCN*) gene [94,95]. Various mutations (over 150) spanning the entire *FLCN* region were observed at the level of the *FLCN* gene, including insertion/deletion, nonsense, missense and splice-site mutations, and partial deletions [96]. The majority of *FLCN* mutations identified in the germline of BHD patients are frameshift mutations (insertion/deletion), nonsense mutations that are predicted to truncate and to inactivate the FLCN protein [96].

FLCN behaves as a classical tumor suppressor gene. These conclusions were supported by a study carried out by Vocke and coworkers on 77 renal tumors derived from 12 patients with germline *FLCN* mutations to identify somatic mutations in the second copy of BHD, showing *FLCN* somatic mutations in 53% of cases and loss-of-heterozygosity at the *BHD* locus in 17% of cases [97]. These findings strongly support the view that *FLCN* gene acts as a tumor suppressor of renal tumorigenesis and both copies of the gene need to be altered for renal cancer development [97].

The study of some germline missense mutations in the folliculin gene, such as *H255Y* and *K508R*, observed in BHD patients with renal carcinomas has directly supported their pathogenic role: the *FLCN H255Y* mutant protein displayed a loss of its tumor suppressive function inducing kidney cell proliferation and the clinical manifestations of BHD, the *FLCN K508R* mutant protein exerted a dominant negative effect on the function of *WT FLCN* in the regulation of kidney cell proliferation [98]. 

Some studies explored the cytogenetic features of these tumors. BHD-associated RCCs, either of chromophobe or of hybrid oncocytic/chromophobe subtype are characterized by a disomic pattern on FISH analysis using probes targeting the centromeric regions of chromosomes 2, 6, and 17, whereas sporadic chromophobe RCCs very frequently displayed a monosomic pattern [99]. Hasumi et al. performed a detailed analysis of the molecular characteristics observed in 29 BHD-associated kidney cancers from 15 BHD patients [100]. All patients displayed *FLCN* germline mutations; somatic *FLCN* mutations were observed in 25 out of the 29 kidney tumors: 20 tumors displayed frameshift/nonsense mutations or loss of heterozygosity at the level of the allele not affected by the germline mutation [100]. Copy number variation in BHD-associated kidney cancer was usually low and was lower in chromophobe and in HOCT histological subtypes than CCRCC and PRCC subtypes; interestingly, in CCRCC subtypes no loss of chromosome *3p* was observed, a condition usually found in sporadic CCRCC [100]. The number of somatic variants was similar in the various histological subtypes of BHD-associated kidney tumors; the frequency of gene mutations was usually low in these tumors, with variants in chromatin remodeling genes being frequently observed (59% of cases); furthermore, variants in genes associated with the mitochondrial pathway, lipid metabolism, and glycolytic pathway were observed in 28%, 24%, and 7% of cases, respectively [100]. Therefore, this study clearly showed that BHD-related renal cancer lacks the mutations in driver genes, such as *TP53, CDKN2A, RB1, PTEN,* and *mTOR*, typically observed in CHRCC. It is of interest to note that at molecular level BHD-related hybrid oncocytic/chromophobe tumors can be differentiated from the sporadic counterpart of these tumors in that these last tumors have copy number losses in chromosomes 1 and XY, but lacks recurrent mutations [101].

The understanding of the molecular mechanisms underlying the BHD syndrome requires the elucidation of the function of *FLCN* gene. The protein folliculin is involved in numerous biological processes, such as membrane trafficking, energy and nutrient homeostasis, and lysosomal biogenesis, and the mutations affecting this protein generate different phenotypes, in relation with their cellular context. FLCN forms molecular complexes with two large proteins, called folliculin interacting protein 1 (FNIP1) and folliculin interacting protein 2 (FNIP2) [102,103,104]. Structural studies have clarified the molecular mechanism induced by FLCN through interaction with FNIP1 and FNIP2: both FLNC and FNIP proteins contain a longin and are differentially expressed in normal versus neoplastic cells (DENN) domain, which are protein folds that have been implicated in the regulation of small GTPases and membrane trafficking [105,106].

Functional studies show that FLCN regulates both the Rag and Rab GTPases depending on nutrient-availability, which are respectively involved in the mTORC1 pathway and lysosomal positioning. Thus, functional studies have shown that *FNIP1* and *FNIP2* act as tumor suppressors since mice deficient in *FNIP1* and *FNIP2* tumors display tumors developing at the level of several organs [107]. Importantly, *FNIP1* and *FNIP2* were essential also for the tumor suppressive function of *FLCN* at the level of kidney tissue, thus supporting the view that the development of kidney tumors in BHD patients may be due to the loss of essential FLCN-FNIP interactions [107]. 

Functional studies support a major role for FLCN-FNIP complex in the regulation of both the Rag and Rab GTPase families, which in turn modulate the mTORC1 signaling pathway and lysosomal distribution, respectively, in a manner dependent upon amino acid availability. mTORC1 is a central, key regulator of cellular metabolism, ensuring cell growth only under suitable conditions [108]. Studies in mice with the kidney-targeted *FLCN* inactivation develop polycystic kidneys and cystic tumors, exhibiting activation of mTORC1 [109,110,111]. Homozygous deletion of FLCN in mice resulted in early embryonic lethality; FLCN heterozygous knockout (*FLCN^+/−^*) mice appeared normal at birth, but developed kidney cysts and solid tumors, as they aged, of different histologic types (oncocytic hybrid, oncocytoma, and clear cell carcinoma with concomitant loss of heterozygosity of *FLCN*); these tumors displayed increased mTORC1 and TORC2 activity [112]. The investigation of other mouse models further supported a role for FLCN as a positive regulator of TORC1 and provided evidence that inappropriate mTORC1 levels can be associated with renal cancerogenesis [113,114]. Interestingly, the tumorigenic potential of FLCN-deficient renal cancer cells is inhibited by sirolimus, a mTOR inhibitor [115].

Recent studies support a functional role for the FLCN-FNIP complex as a GTPase activating protein involved in the fine modulation of Rag GTPase are nucleotide binding and transmission of the nutrient status to mTORC1 [116]. It was proposed that the GTPase activating properties of the FLCN-FNIP complex occurs downstream of GATAR1 protein complex and together orchestrate a unique molecular regulation: when amino acid levels are low, the GTPase activating protein activity of GATOR1 promotes the GDP-Rag A7B condition and the FLCN-FNIP complex is recruited at the level of lysosomes to drive the GTPase activating properties toward Rag C/D [117,118].

FNIP1 and FNIP2 were also identified as proteins capable to interact also with AMPK, although FLNC does not seem to be essential for FNIP-AMPK interaction [102,103,104]. AMPK is a heterotrimeric kinase whose activation increases ATP production through stimulation of catabolic pathways, concomitantly with the inhibition of anabolic pathways that consume ATP, in a way antagonistic to mTORC1 activity. Furthermore, various studies have shown that FLCN deficiency triggers AMPK activation [119,120,121]; furthermore, *FNIP1* mutations are associated with high AMPK activity [122].

Finally, FLCN deficiency exerts also important effects at the level of energetic metabolism, with a consistent metabolic change in favor of aerobic glycolysis. Thus, Yan et al. reported a “Warburg effect” metabolic transformation in FLCN-deficient embryonic fibroblasts, with increased glucose uptake, lactate production, and extracellular acidification, associated with HIF transcriptional activity and enhanced expression of HIF-dependent genes [120]. The increase in metabolic activity was associated in FLCN-deficient fibroblasts with an increased mitochondrial mass and respiration [120]. This effect elicited by FLCN deficiency on mitochondrial mass is remarkable and seems to be associated with an enhanced expression and activity of PGC1α (peroxisome proliferation-activated receptor gamma coactivator 1-alpha), a transcriptional regulator of genes involved in mitochondrial biosynthesis [123]. PGC1α levels were found to be elevated in FLCN-deficient renal cancers [124]. Furthermore, BHD-related tumors were characterized by up-regulation of mitochondrial gene expression [124]. The study of FLCN-deficient mice clearly showed the existence of a condition of chronic AMPK activation, which in turn, induces the expression and activation of PGC1α [125].

## 9. Familial MITF Microphtalmia-Associated Transcription Factor

Subjects carrying a germline pathogenic variant of *MITF* have a more than five-fold increased risk of developing melanoma and renal cancer, as compared to the individuals not bearing these variants. The molecular characterization of these *MITF* oncogenic variants showed a mutation at the level of codon 318 (E318K), located at the level of a small-ubiquitin-like modifier (SUMO) consensus site (ψKXE), determining a strong impairment of SUMOylation of MITF [126]. The E318K mutation increased the binding to the HIF1α promoter and increased its transcriptional activity [126]. However, the *MITF E318K* mutation does not seem to be involved in sporadic RCC: in fact, in a screening based on the analysis of 403 sporadic RCCs only one *MITF E318K* mutation was detected [127].

## 10. Chromophobe Renal Cancer

CHRCC is the second most common form of non-CCRCC after papillary RCC and displays a frequency corresponding to 5–10% of all RCCs. The main molecular features of CCRCC are reported in Table 2. The analysis of the genomic alterations observed in CHRCC supports that this tumor subtype originates from distal convoluted tubules, compared with other kidney cancers, including CCRCC with more origin from proximal tubules [128].

The main symptoms of patients with CHRCC at presentation are represented by flank pain and hematuria. CHRCCs are usually solitary tumors that can reach a big size (up to 25 cm in diameter). At microscopic level, these tumors are usually arranged in solid sheets, with tumor parenchyma intersected by fibrous septa and blood vessels. Two cellular elements usually compose these tumors: one, chromophobe cells, being represented by large polygonal cells with abundant, chromophobe cytoplasm and the other one represented by smaller cells, with a small eosinophilic cytoplasm. It was described a variant of chromophobe RCC described as eosinophilic variant of CHRCC and characterized by the whole composition by intensively eosinophilic cells; two types of cellular elements have been described in these tumors: type 1 cells, small with moderately granular cytoplasm and type 2 cells, with abundant eosinophilic cytoplasm denser at the periphery [129]. The genetic abnormality most frequently observed in CHRCC is represented by the loss of one copy of the entire chromosomes *1, 2, 6, 10, 13, 17,* and *21* (observed in about 86% of cases) and losses of several other chromosomes (observed in about 12–58 of cases) [130]. These chromosome abnormalities have been observed both in the classic and in the eosinophilic variants of CHRCC, although loss of chromosomes *2* and *6* was less frequent in eosinophilic than in classic variant of CHRCC [131]. It is important to note that about 50% of CHRCCs display loss of all chromosomes and about 10% display no loss of any chromosome [131]. The chromosome losses were not observed in oncocytomas [131]. A recent study, through cumulative analysis of various database containing data on chromosome number alterations in CHRCC, reached the conclusion that losses of chromosomes *1, 2, 6, 10, 13,* and *17* were significantly more frequent among classic CHRCC compared to eosinophilic CHRCC, thus suggesting that classic CHRCCs are characterized by higher chromosomal instability [132]. In addition to these typical chromosomal losses, CHRCCs display also copy number gains that were detected in chromosomes *4, 7, 11, 12, 14q,* and *18q* [133].

About 2% of CHRCC display sarcomatoid features; these tumors were explored for their chromosomal abnormalities, showing some remarkable differences compared to the rest of CHRCC: sarcomatoid CHRCCs frequently display multiple gains (polysomy) of chromosomes *1, 2, 6, 10,* and *17*; distant metastases show the same chromosome abnormalities, usually chromosome losses found in the primary tumors [134].

The analysis of gene copy number by next generation sequencing showed the occurrence of multiple abnormalities in CHRCC; this analysis showed that the two most frequent deletions involved the tumor suppressor genes *RB1* and *ERBB4* [135]. Fluorescence in situ hybridization showed hemizygous deletion of *RB1* in 52% of cases and of *ERBB4* in 33% of cases; in total, 70% of CHRCC display either hemizygous deletion of *RB1* or *ERBB4* [135].

Davis and coworkers in the context of TCGA studies have performed a comprehensive characterization of 66 primary CHRCC using diverse molecular platforms, including whole-genome sequencing and mtDNA analysis [128]. The results of this study showed: (i) The typical and frequent chromosome losses described in other studies, observed in all cases corresponding to the classic variant and in about 53% of cases corresponding to the eosinophilic variant; (ii) *TP53* (32% of cases) and *PTEN* (9% of cases) were the only two genes frequently mutated in these tumors, while mutations of other cancer-relevant genes (such as *MTOR, NRAS*) were found at lower frequencies; (iii) the gene expression profile showed a high index of mRNA expression correlation for CHRCC with distal regions of the nephron; (iv) the analysis of mitochondrial DNA showed mutations at the level of genes involved in respiration and oxidative phosphorylation; (v) whole genome sequencing analysis showed the occurrence of kataegis (a mutational phenomenon involving highly localized substitution mutations, C > T or C > G), occurring at the level of some chromosome regions involved in rearrangements, involving also rearrangements occurring within the *TERT* promoter gene region (observed in 12% of cases) and associated with elevated TERT expression [128]. 

Durinck et al. have reported a study of extensive characterization of the genomic alterations observed in non-clear RCC subtypes, including CHRCC (36 classic and 12 eosinophilic). In CHRCC, the frequently mutated genes were: *TP53* (21.3%); *PTEN* and *KIAA* 1731 (6.4% of cases); *FAAH2, PDHB, PDXD1, ZNF 765, PRKAG2, ARID1A,* and *ABHD3* (4.3% of cases) [136]. Some of these mutations may play a relevant role in the pathogenesis of CHRCC. Thus, the *PDHB* gene encodes the E1β subunit of the pyruvate dehydrogenase complex (PDHc), catalyzing the conversion of pyruvate to acetyl-CoA, thus providing a link between glycolysis and the TCA cycle; the two mutations observed in CHRCC are reminiscent of those observed in a neurological condition associated with germline mutations of this gene and causing lactic acidosis [136]. *PRKAG2* encodes one of the three γ subunits of AMPK, a key sensor of cellular metabolism; the mutations of this gene, observed at the level of the inhibitory pseudosubstrate sequence within AMPK γ subunit, may lead to constitutive AMPK activation [136]. Furthermore, gene expression analysis led to the identification of five genes, *ADAP1, SDCBP2, HOOK2, BAIAP3,* and *SPINT1* markedly expressed in CHRCCs and that clearly differentiated these tumors from oncocytomas [136]. 

Ricketts et al. reported a comprehensive analysis of different subtypes of RCCs, including 81 cases of CHRCC [137]. Some recurrent mutations have a prognostic impact in CHRCC patients: *PTEN* mutations correlated with decreased survival; *CDKN2A* alterations (including loss of the region of chromosome 9p encoding *CDKN2A* and promoter hypermethylation) correlated with a decreased survival [137]. About 20% of CHRCCs displayed a hypermethylation DNA profile and these tumors were associated with a higher tumor grade and with a poor outcome [137]. The metabolic gene expression profile showed that: expression of the Krebs cycle and the electron transport chain genes was high in CHRCC, in association with increased expression of the pyruvate dehydrogenase complex activation genes; expression of AMPK was increased in CHRCC; a small subgroup of CHRCCs displayed a peculiar metabolic profile with low expression of the Krebs cycle and electron transport chain genes, lower expression of the *AMPK* pathway genes, and increased expression of the genes in ribose synthesis pathway, and was associated with a particularly poor prognosis [137].

Although CHRCC is a relatively indolent tumor, 5–10% of patients may develop metastases and metastatic tumors may possess peculiar molecular properties compared to those not generating metastases. This analysis provided evidence that metastatic CHRCC, at variance with non-metastatic CHRCCs that are hyperdiploid (with a ploidy estimated above 2); importantly, these hyperdiploid metastatic CHRCC maintained their typical CHRCC-*7* set- chromosomes loss [138]. This hyperdiploid pattern is due to either loss of the CHRCC-*7* set-chromosomes, associated with duplication of the remaining genome or duplication of multiple chromosomes excluding the CHRCC-*7* set-chromosomes: this condition was defined as imbalanced chromosome duplication (ICD) [138]. The comparative analysis of metastatic and non-metastatic CHRCC showed among metastatic tumors increased frequencies of *TP53* mutations, *PTEN* mutations, and ICD (observed at frequency of 55%, 27%, and 43%, respectively) compared with those observed in nonmetastatic CHRCC (25%, 7%, and 10%, respectively) [138]. Phylogenetic studies of paired-primary-metastatic samples allowed to propose a tumor progression process, involving the nearly universal loss of CHRCC-7 set-chromosomes as the only driver event in the pathogenesis of CHRCC, followed by *TP53* mutations that were detected in 82% of metastatic samples and then by ICD and *PTEN* mutations that were detected in 82% of metastatic samples and then by ICD and *PTEN* mutation, occurring in a mutually exclusive manner [138].

Initial studies have shown that the membrane receptor KIUT is overexpressed in CHRCC (83% of cases positive), whereas it was not expressed in other RCCs [139].

CHRCC is usually associated with a favorable prognosis. Przybycin and coworkers in a retrospective study in 200 CHRCC patients have shown that: 2.5% of cases displayed metastases at presentation; disease-specific events, including recurrence, metastasis, and death due to disease were observed in additional 4% of patients; 2% of patients had tumors with sarcomatoid features [140]. 5-year and 10-year disease-specific events occurred in 3.7% and 6.4% of patients, respectively [140]. Therefore, these observations showed a significant association of outcomes with tumor size; small-vessel invasion, sarcomatoid features, and microscopic necrosis, whereas T stage showed a statistically non-significant association [140]. A large multicenter study involving the analysis of 291 patients with CHRCC diagnosis confirmed the good prognosis of CHRCC patients, with only 1.3% of these patients presenting distant metastases at diagnosis and a 5-year and 10-year cancer-specific survival of 93% and 88.9%, respectively [141]. Only patients with locally advanced disease at diagnosis or with metastatic cancers, as well as those with sarcomatoid differentiation have a poor prognosis [141].

Because of the rarity of this condition, only few studies have specifically explored the outcomes of metastatic CHRCC patients. The analysis of a very large cohort of 4970 metastatic RCC patients treated with targeted therapy showed that only 2.2% of these patients displayed metastatic CHRCC and the large majority (97.8%) pertains to CCRCC [142]. Metastatic CHRCC exhibited a similar overall survival compared to patients with CCRCC (23.8 months vs. 22.4 months) [142]. Ged and coworkers have analyzed the outcomes of metastatic CHRCC according to the presence or not of sarcomatoid features [143]. In a group of 109 metastatic CHRCC patients, these authors observed that 29 of them exhibited sarcomatoid differentiation; patients with sarcomatoid features showed a shorter time to metastatic recurrence than those with non-sarcomatoid differentiation (2.7 months vs. 48.8 months); a similar observation was made for time to treatment failure (1.8 months vs. 8.0 months). Finally and importantly, median overall survival was clearly inferior for patients with sarcomatoid differentiation compared to those without this differentiation properties (7.5 months vs. 38 months) [143]. Recently, Casuscelli et al. have reported a survey on a very large cohort of 496 CHRCC patients diagnosed and surgically treated at Memorial Sloan Kettering Cancer Center [144]. This study definitely confirmed the findings observed in previous studies, showing that: at 10 years, the relapse-free survival was 91.7% and the overall survival 82.1% for CHRCC patients, compared to 79.4% and 63.6% for CCRCC patients; patients with CHRCC displayed less frequently sarcomatoid differentiation compared to CCRCC patients (1.2% vs. 4%); larger tumor size, sarcomatoid differentiation, and higher tumor-stage are significantly associated with adverse RFS and OS in CCRCCs [144].

## 11. Papillary Renal Carcinoma

PRCCs make up about 15% of RCCs, are heterogeneous, and characterized by the presence of papillae in the tumor; these tumors are commonly subdivided into two subtypes based on staining features: subtype 1 basophilic, type 2 eosinophilic [145]. Particularly, type 1 PRCCs display papillae lined by a single layer of cells with scanty basophilic cytoplasm and low nuclear grade; type 2 PRCCs show papillae lined by pseudostratified layers of cells with more abundant eosinophilic cytoplasm and low nuclear grade [145]. About 15% of PRCCs cannot be classified as type 1 or type 2 subtypes and are grouped in an unclassified group.

Type 1 and type 2 PRCCs have distinct molecular pathways and clinical behavior. Type 2 tumors were larger, more common in patients younger than age 40, and more frequently stages 3 or 4 than were type 1 tumors [146].

In 2016 TCGA provided the first detailed, comprehensive molecular analysis of PRCCs [147]. The study of copy number alterations displayed the existence of three main tumor subgroups: (i) One subgroup is predominantly composed of type 1 and lower-grade tumors and is characterized by multiple chromosomal gains involving the very frequent gain of chromosomes *7* and *17* and the less frequent gain of chromosomes *2, 3, 12, 16,* and *20*; (ii) the other two subgroups are predominantly composed by type 2 tumors and one of these two subgroups is characterized by a limited number of copy alterations, whereas the other one is characterized by extensive aneuploidy, with numerous chromosomal losses, including frequent loss of chromosome *9p* and is associated with poor survival [147]. The higher frequency of the number of DNA gains per tumor at the level of chromosomes *7* and *17* in type 1 than in type 2 PRCCs was previously reported by Jiang and coworkers [148].

Whole exome sequencing performed in 157 PRCCs identified several somatic mutations, occurring with a significant frequency, at the level of tumor-related genes, such as *MET, SETD2, NF2, KDM6A, SMARCB1, FAT1, BAP1, PBRM1, STAG2, NFE2L2,* and *TP53* [63,147]. Assignment of these genes to their biochemical pathways showed that: SWI/SNF complex (*SMARC1* and *PBRM1*) was altered in 20% of type 1 and 27% of type 2 PRCCs; chromatin modifier pathways (*SETD2, KDM6A,* and *BAP1*) was altered in 35% of type 1 and 38% of type 2 PRCCs; the Hippo pathway (*NF2*) was altered in 3% of type 1 and 10% of type 2 PRCCs [63,147]. However, some genetic alterations are specific to types of PRCCs: (1) *MET* mutations are much more frequent in type 1 than type 2 PRCCs (17% vs. 1.6%, respectively) and were observed in 11% of unclassified PRCCs; levels of MET mRNA and MET protein phosphorylation were higher in type 1 than type 2 tumors. (2) 8% type 2 PRCCs displayed *9p21* chromosomal focal loss with loss of *CDKN2A* locus; other type 2 PRCCs exhibited *CDKN2A* mutations or promoter hypermethylation, resulting in a total of 13% of tumors with *CDKN2A* alterations; *CDKN2A* loss was associated with low overall survival. (3) Type 2 PRCCs are associated with mutations in chromatin-modifying genes *SETD2* (19.4%), *BAP1* (10.4%), and *PBRM1* (11.9%) which are frequently mutated in CCRCCs; mutations of *BAP1* and *PBRM1* were mutually exclusive, whereas *SETD2* mutations co-occurred with PBMR1 mutations in most cases. (4) Another feature of type 2 PRCCs consists in the increased expression of NRF2-associated response element (ARE) pathway; these findings were in line with other studies showing increased activation of the NRF2-ARE pathway in type 2 PRCCs and mutations in NRF2-ARE pathway genes *NFE2L2, CUL3, KEAP1,* and *SRT1* [82,84,149,150]. (5) A CpG island methylator phenotype (CIMP) was observed in a subgroup of type 2 PRCCs characterized by mutations of *FH* gene and poor survival [63,147]. 

Finally, from this study it emerges that unclassified PRCCs display molecular properties hybrid between type 1 and type 2 PRCCs; the frequency of chromosomal 7 gain in these tumors is intermediate (26%) between type 1 (85%) and type 2 (18%) [63,147].

Durinck et al. in their study of molecular characterization of non-clear RCCs reported a detailed analysis of *MET* mutations occurring in PRCCs; particularly, they observed *MET* mutations in 15% of the PRCC samples: all these mutations, with just a single exception, affected the kinase domain of MET, all displaying elevated phosphorylation, suggesting their constitutive activation [136].

A large data set of 169 patients with advanced PRCC was published by Pal et al., basically corroborating the data reported in the TCGA study [151]. Particularly, in patients with type 1 PRCC the most commonly altered genes were *MET* (33%), *TERT* (30%), *CDKN2A/B* (18%), and *EGFR* (8%); in patients with type 2 PRCC the most recurrent gene mutations were *CDKN2A/B* (18%), *TERT* (18%), *NF2* (13%), *FH* (13%), and *MET* (7%) [151]. Remarkable differences from TCGA data involve higher frequencies of *MET, NF2,* and *CDKN2A/B* [151].

In 2018, TCGA network refined the molecular analysis of PRCCs, showing that: at the level of single gene mutations, in PRCCs *TP53* and *PBRM1* mutations correlated with decreased survival; *CDKN2A* mutation, hypermethylation, or deletion was found in 5% of type 1 PRCC, 18.6% of type 2 PRCC, 100% of CIMP-PRCC, and was associated with decreased survival; at the level of DNA methylation analysis, increased hypermethylation was associated with higher-stage disease in both type 1 and type 2 PRCCs and with decreased survival: among the hypermethylated genes, there were WNT pathway regulatory genes *SFRP1* and *DKK1*, whose hypermethylation was associated with poor survival; at the level of metabolic gene expression features, type 2 PRCCs displayed a more elevated expression of Krebs cycle genes compared to type 1 PRCC; concerning the immune signature analysis, both in whole population of PRCC and in type 2 PRCC, the high expression of a high T helper 2 (Th2) was associated with a reduced survival [137].

Two studies have reported the characterization of PRCCs by whole-genome sequencing. Li and coworkers using this approach discovered mutations at the level of an intron of *MET* gene, connected to an oncogenically relevant splicing event; furthermore, in other cases a methylation dysregulation on nearby, leading to a cryptic promoter activation of the *MET* gene was identified [152]. Furthermore, it was identified the recurrent mutation of the long noncoding RNA *NEAT1* and these mutations are associated with increased *NEAT1* expression and negative outcome [152]. Zhu and coworkers have explored the intratumoral heterogeneity and clonal evolution of PRCC integrating whole-genome sequencing and DNA methylation data [153]. Through the analysis of 29 patients at the level of various tumor regions (center and periphery of each tumor) the authors reached the important conclusion that, at variance with previous studies in CCRCC, in PRCC driver gene mutations and most arm-level somatic copy number alterations are clonal [153].

The main treatments used for RCC patients are based on clinical studies involving a limited participation from patients with PRCC; therefore, it is not surprising that conventional therapies are usually less for non-CCRCC compared to CCRCC. Thus, PRCCs are less responsive to conventional therapy used in RCC compared with CCRCCs, both at the level of PFS and OS, as supported by the analysis of large cohorts of patients [154,155]. The same difference in therapeutic response applies to VEGF inhibitors, such as sunitinib by showing shorter PFS in metastatic PRCC compared to metastatic CCRCC. However, sunitinib treatment in metastatic PRCC induced a slightly better PFS compared to the mTOR inhibitor everolimus and this gives support to the choice of the guidelines from the National Comprehensive Cancer Network and the European Society for Medical Oncology both recommending sunitinib as first line therapy in metastatic non-CCRCC. 

Various agents targeting MET, such as crizotinib, savotinib, cabozantinib, foretinib, and tivantinib have been explored in clinical trials involving PRCC patients [156].

Among the studies carried out with MET inhibitors promising are those with cabozantinib and savolitinib. Two retrospective studies have shown therapeutic activity of cabozantinib in PRCC patients [157,158]. In fact, both these studies showed an objective response rate in metastatic PRCC patients treated with cabozantinib ranging from 14% to 27%, with a mean overall survival of 11 months in one of these studies [157,158]. Recently, the results of the SAVOIR phase 3 randomized clinical trial, comparing the efficacy of savolitinib to sunitinib in patients with MET-driven PRCC were published: in this study, a PFS of 7.0 months for savolitinib and of 5.6 months for sunitinib was observed, with significantly fewer adverse events reported in the savolitinib arm compared to the sunitinib arm [159]. These results suggest that savolitinib shows an encouraging efficacy compared to sunitinib. 

Finally, another recent clinical study explored the association of a MET inhibitor (savolitinib) with a PD-L1 inhibitor (durvalumab). The first results observed in the PRCC cohort of the phase I/II CALYPSO clinical trial were recently presented at the ASCO Meeting [160]. In a population of PRCC patients with metastatic PRCC either treatment-naïve or VEGFR TKI-resistant, an OS at 12 months of 52%, not showing significant differences among PD-L1 positive, MET positive, and PD-L1/MET negative patients, was reported [160]. Some patients displayed durable responses [160].

## 12. Genetic Alterations of CCRCC

The most frequent and typical genetic alteration of CCRCC is represented by biallelic inactivation on the *VHL* gene determined by allelic deletion or loss of heterogeneity on chromosome *3p* (observed in >90% of cases) [161], together with gene mutation (observed in about 50% of cases) [162,163] or promoter hypermethylation (observed in 5–10% of cases) [164]. Other frequent genetic alterations are represented by mutations in genes involved in chromatin modification, such as *PBRM1* [165], *SETD2* [166], *KDM5C* [166], *KDM6A* [166], and *BAP1* [167,168].

Sato and coworkers reported the first comprehensive, integrated molecular analysis of CCRCC [169]. Four (*VHL, PBMR1, SETD2,* and *BPA1*) of the five mutated genes in CCRCC are all located at the level of the 3p chromosomal region involved in LOH; 98% of the CRCC cases displaying LOH at 3p showed the remaining *VHL* allele altered by somatic mutation or promoter methylation [169]. Almost all cases exhibiting *PBMR1, SETD2,* and *BAP1* mutations occurred in CCRCC cases displaying *VHL* inactivation. Importantly, *SETD2* and *BAP1* mutations displayed lower allelic burdens than coexisting *VHL* mutations, suggesting that these mutations are acquired at later times during tumor development [169]. *PBRM1* mutations had no significant impact on overall survival, whereas *BAP1* mutations, mutually exclusive with *PBRM1* mutations, were associated with a shorter overall survival; finally, *SETD2* mutations displayed a high relapse rate [169]. Interestingly, 5% of CCRCC patients displayed *TCEB1* mutations, not associated with *VHL* gene alterations, but constantly associated with loss of chromosome 8; *TCEB1* encodes a protein involved in the formation of the RNA polymerase II elongation factor complex but also involved in the VHL complex formation [169]. In line with this finding, *TCEB1*-mutated tumors displayed increased HIF-1αprotein expression, as well as tumors with VHL loss [169]. Therefore, CCRCC with VHL loss or with *TCEB1* mutations accounts for 95.4% of the cases. Other genes recurrently mutated in CCRCC included *TET2, KEAP1,* and *MTOR*: *TET2* mutations and deletions occurred in 16% of cases; mutually exclusive mutations in *KEAP1, NRF2,* and *CUL3* occurred in 6.6% of cases; *MTOR* mutations were observed in 5.7% of cases [169]. 

A parallel study by TCGA reported the comprehensive molecular analysis of 417 samples of CRCC [170]. Most of the results reported in this analysis are in line with those reported by Sato et al. [169] and here are discussed the results of this study not analyzed in the other study. At the level of copy number the most recurrent event was loss of chromosome *3p* observed in 91% of cases; *17q* chromosome loss, associated with loss of *HIF1A* and with a more aggressive disease, was observed in 45% of samples; gains of *5q* were frequently observed (67% of cases); several focal amplifications involved genes relevant at oncogenic level, such as *PRKC1, MDS1, EVI1, MDM4, MYC, JAK2*; focally deleted regions involved the tumor suppressor gene *CDKN2A* and *PTEN* [170]. Importantly, Sato et al. reported among the CNAs the loss of *8p* with or without loss of *8q* (20% of cases), an abnormality frequently associated with *TCEB1* mutations [169]. Integrative data analysis showed that the most frequently mutated network involved VHL and numerous interacting partners, leading to activation of the transcription factor program mediated by HIF1A/ARNT; the second most mutated network included *PBRM1, ARID1A,* and *SMARCA4*, key genes at the level of chromatin remodeling complex; the mutations of the chromatin regulators *PBRM1, STD2,* and *BAP1* induce different patterns of altered gene expression in the context of a background caused by VHL loss; mutually exclusive alterations targeting multiple complexes of the PI3K/AKT/MTOR pathway occur in about 28% of the cases and suggest a potential therapeutic targeting [170]. In their evaluation of the main signaling pathways, Sato et al. evaluated all genetic alterations occurring in CCRCCC-inducing activation of PI3K signaling and estimated a frequency of 76% of cases exhibiting PI3K activation; furthermore, they reported also the frequent (40%) activation of p53 signaling [169].

Finally, the TCGA study reported clear evidence about a metabolic gene expression pattern associated with aggressive disease, related to downregulation of genes the pentose phosphate pathway and the glutamine transporter genes and increased acetyl-CoA carboxylase protein levels [170].

The focal amplifications occurring at the level of chromosome *5q* were explored in greater detail in subsequent studies. Copy number gains of chromosome 5q occurring in CCRCC drive overexpression of the gene *SQSTM1*; the p62 SQSMT1 protein is involved in activation of NRF2, and through this mechanism, in promotion of resistance to redox stress and in stimulation of renal cancer cell growth in vitro and in vivo [171]. A study based on multi-region whole-genome sequencing of 30 CCRCCs in the context of the TRACERx study showed that the gain of the chromosome arm *5q*, together with the loss of chromosome arm *3p* occur at the same time during CCRCC development: the concomitant occurrence of these two chromosomal abnormalities may be mediated by an unbalanced translocation event occurring between chromosomes 3 and 5 that involves chromotripsis [18,172]. This event was proposed as the initiating event for CCRCCs [18,172].

Ricketts et al. refined the analysis of molecular abnormalities of CCRCC performed by TCGA and showed that in these tumors: *TP53* and *BAP1* mutations and CDKN2A alterations were associated with decreased survival; at mRNA expression level an increased expression of the vasculature development signature, due to the activation of the VHL/HIF pathway, increased the immune response signature compared to other RCC types and increased ribose metabolism pathway, associated with poor survival [136].

Few studies have investigated the genomic landscape of metastases compared to primary tumors in CCRCC. At histological level, metastatic CCRCC tumors display pathological features similar to those of primary tumors from which they derive [173]. At gene expression level, the paired analysis of primary and metastatic CCRCC displayed an enrichment in metastatic tumors of the expression of genes involved in the formation of extracellular matrix [174]. De Velasco and coworkers have reported the analysis of a large cohort of metastatic CCRCCs, and through the analysis of matched metastases and primary tumors reached the conclusion that CCRCC primary tumors and metastases display a highly comparable distribution of common genetic alterations [175]. This finding supports the view that there is no single gene driving the metastatic disease or that changes at expression protein or epigenetic level are responsible for the development of metastatic properties of CCRCCs [175].

The study of tumor heterogeneity provided more information in the understanding of the molecular mechanisms involved in CCRCC evolution. In this context, fundamental were two studies by Gerlinger and coworkers reporting the analysis of 10 CCRCC patients (7 with metastatic disease) by exome sequencing on multiple regions of the same tumor and performing a comparison with a mutational spectrum across all regions [176,177]. The results of these two studies provided some fundamental data about intratumor heterogeneity of CCRCC: only a small fraction of genetic alterations display a clonal distribution, such as VHL loss and chromosome arm 3p loss, whereas other genes recurrently mutated such as *SETD2* and *BAP1* have a subclonal pattern of distribution within the tumor [176,177]. It is of interest to note that the multi-region sequencing allowed the identification of a higher frequency of gene mutations and copy number alterations than by single tumor sampling [176,177]. Thus, according to these data it is possible to infer that the TCGA data obtained on single tumor sampling could underestimate the frequency of some driver mutations such as *BAP1* and *TP53* [176,177].

These initial observations have been expanded through the multiregional analysis of 100 primary CCRCC and 38 cases of metastases; these two additional studies strongly supported the view that the intertumor heterogeneity and the pattern of intratumor heterogeneity influence the tumor evolution and metastasis development [178,179] (Table 3). Particularly, variations in the number, timing, and order of driver events are major determinants of disease evolution and metastatic potential. In tumors in which VHL is the only driver event, metastatic evolution is rare, whereas cases with multiple drivers are associated with metastatic development; the sequence and the intratumor distribution of these additional drivers, either clonal (present in all tumor cells) or subclonal (present in all tumor cells) or subclonal (present in only a part of tumor cells) is a key determinant of tumor evolution, thus if the driver events in addition to VHL loss occur clonally the metastatic spread is slower [178]. Thus, CCRCC characterized by low chromosomal complexity and low intratumor heterogeneity evolves following a linear pathway with *VHL* as sole mutation; CCRCC evolving through a branched pathway acquires early *PBRM1* mutation and subsequent subclonal driver alterations slowly evolves to a oligometastatic potential; CCRCC evolving through a punctuated pathway results from the development of tumors characterized by the presence of multiple driver genetic alterations occurring clonally (punctuated evolution) and evolves more rapidly to metastatic potential [178] (Table 3). These studies showed also that losses of chromosomes *9p* and *14q* are events of fundamental importance for metastatic evolution of CCRCC: these two chromosomal abnormalities are enriched in all metastases and are therefore drivers of metastatic progression and higher overall mortality [179].

Huang and coworkers have analyzed the clonal architectures of 473 CCRCC patients and showed that the evolution patterns of CCRCC have consistent inter-patient heterogeneity, with del(*3p*) being considered as the common earliest molecular event, followed by three most recurrent patterns of clonal evolution dictated by different molecular events: (i) *VHL* and *PBRM1* mutations; (ii) del(*14q*); (iii) amp(*7*), del(*1p*), del(*6q*), amp(*7q*), del(*3q*) [180]. The analysis of these patients allowed to identify three prognostic subtypes of CCRCC with different clonal architectures and immune infiltrates: patients with a long-life expectancy are enriched with VHL, but depleted of *BAP1* mutations, and have high levels of Th17 and CD8^+^T lymphocytes, while patients with a short survival are characterized by high burden of CNAs (frequent del(*14q*)), high levels of Tregs and Th2 cells [180].

Recently, Clark and coworkers reported an integrated proteogenomic characterization of CCRCC; in this study, 110 treatment-naïve CCRCCs were explored by wide genome sequencing and by epigenomic, transcriptomic, proteomic, and phosphoproteomic analyses. At arm level, *3p* loss (93%) was the most frequent CNA, followed by *5q* gain (54%), chromosome *14q* loss (42%), chromosome *7* gain (34%), and chromosome *9* loss (21%); furthermore, about 13% of tumors displayed extensive copy number variations along all chromosomes, thus indicating a high degree of genomic instability [181]. This analysis showed also that 61% of CCRCC cases displayed one or more translocations, mainly involving the chromosome *3p* locus and chromosome *5* (20%) [181]. This study confirmed the data on the frequency of most recurrence gene mutations and provided evidence that all the genetic alterations, including *VHL, PBMR1, BAP1, KDM5C,* and *SETD2* are related to genetic events resulting in reduced expression of both mRNA and protein, thus indicating loss-of-function and supporting the classification of these genes as tumor suppressors [181]. The proteomic analysis allowed to better characterize the metabolic shift occurring in CCRCC tumors, illustrated at protein level by upregulation of glycolysis and downregulation of the Krebs cycle and electron transport chain (OXPHOS), associated with the Warburg effect; the downregulation of the Krebs cycle and the majority of OXPHOS proteins were not observed at mRNA level [181]. This analysis of proteo-metabolic profile allowed also to identify late-stage tumors upregulating OXPHOS pathway relative to early stage tumors, a finding that may be related to dysregulation of HIF-1 α caused by 14q loss; similar observations were previously reported by Hakimi et al. through analysis of the metabolic profiling of CCRC [182]. The proteomic analysis allowed the subdivision of CCRCC into three groups: CCRCC1 associated with higher tumor grade and stage and characterized by elevated adaptive immune response, N-linked glycosylation, OXPHOS protein expression and fatty acid metabolism and high frequency of BAP1 mutations and CIMP^+^ status; CCRCC2 and CCRCC3 were associated with lower tumor grade and stage: tumors in CCRCC2 were associated with tumor immunity, whereas tumors in CCRCC3 with glycolysis, mTOR signaling, and hypoxia and display higher frequency of PBRM1 mutations [181].

Over the past years, the therapy for patients with advanced/metastatic CCRCC has considerably evolved and new therapeutic options are now available for these patients, including targeted agents such as those targeting the VEGF pathway (mainly represented by VEGFR-directed tyrosine kinase inhibitors, TKIs) or targeting mTOR (such as everolimus) or immunotherapy based on immune checkpoint inhibitors and combination treatment strategies [183]. Molecular studies have contributed to define the subpopulations of CCRCC patients more responsive to these treatments and to define the mechanisms of primary or acquired resistance to these therapies. 

Thus, several retrospective studies have analyzed the prognostic impact of chromatin-modifying gene alterations in CCRCC. *PBRM1*, the gene most frequently altered after *VHL*, seems to play a different role in localized and advanced disease, constituting a poor prognostic factor in localized disease and a good prognostic factor in advanced disease [184]. Retrospective studies on metastatic CCRCC patients indicate that *PBRM1* loss is associated with improved outcomes in patients treated with either VEGFR TKIs or mTOR inhibitors, whereas *BAP1* and *TP53* mutations were associated with unfavorable cancer-specific outcomes [185]. 

A part of patients with advanced CCRCC respond to treatment with immune check blockage and some of these responses are durable. Immune check inhibitors (ICI) have become a key therapeutic strategy to stimulate the immune anti-cancer response; across various solid tumor malignancies, response to PD-1 or PD-L1 blockade was associated with some tumor-intrinsic (high tumor antigen burden, high neoantigen load) or microenvironmental features (PD-L1 expression, T lymphocyte infiltration). McDermott and coworkers have analyzed the CCRCC patients enrolled in the context of IMmotion150 clinical trial, a randomized phase II study of atezolizumab (anti-PD-L1) alone or in combination with bevacizumab (anti-VEGF) versus sunitinib (multi TKI) [186]. Exploratory biomarker analyses failed to show that tumor mutation burden and neoantigen load display any significant association with PFS; angiogenesis, T-effector/IFN-gamma response, and myeloid inflammatory gene expression signatures were strongly and differentially associated with PFS [186]. 62% of these patients displayed VHL mutations and 44% *PBMR1* mutations; angiogenesis-related gene expression signature was higher in VHL-mutated and *PBRM1*-mutated CCRCCs; within treatment evaluation showed that *PBRM1* mutations were associated with improved PFS in the sunitinib arm; in the *PBRM1*-mutated patients atezolizumab+bevacizumab showed improved PFS compared to atezolizumab alone [186]. Whole genome sequencing studies performed in 35 metastatic CCRCC patients undergoing treatment with an anti-PD-1 blocking agent showed that clinical benefit to this treatment was significantly associated with mutations in the *PBMR1* gene [187]. These findings were confirmed in independent validation cohorts of CCRCC patients treated with PD-1 or PD-L1 blockade therapy [188].

The analysis of 592 tumors derived from patients with advanced CCRCC enrolled in clinical trials based on the treatment with PD-1 confirmed that conventional genomic and immunological markers were not associated with clinical response, but some genomic abnormalities associated with response or resistance to PD-1 blockade [189].

## 13. Genetic Abnormalities of Renal Medullary Carcinoma (RMC)

RMC is a rare aggressive subtype of renal cancer that mainly affects young adults with sickle cell trait. This condition was initially described by Davis et al. in 1995, reporting a series of cases of aggressive kidney cancers occurring in young individuals (15–30 years) with sickle cell trait; most of these patients presented with advanced disease and poor survival [190]. Beyond the strong clinical association with sickle disease trait, the underlying biology of this rare cancer is poorly understood. Loss of *SMARCB1* (also known as INI1) is a key diagnostic feature of these tumors: Calderaro et al. reported the loss of *SMARCB1* expression by immunohistochemistry by 6/6 RMC patients; in two cases explored by FISH analysis, loss of one *SMARCB1* allele was observed [191].

The mechanisms underlying SMARCB1 protein loss in RMC were explored by more recent studies. Thus, in 2016 Calderaro et al. reported novel balanced translocations disrupting *SMARCB1* in 4 of 5 cases studied; all these 4 cases occurred in patients with sickle cell trait or disease, whereas the remaining case displayed a homozygous deletion of *SMARCB1* and presented in a patient with normal hemoglobin [192]. Total of 36 patients with RMC were reported by Carlo et al.; 33 of these patients were explored for tissue expression by immunohistochemistry and 100% of them displayed *SMARCB1* loss; 10 patients were explored by FISH analysis and 2 of them displayed biallelic *SMARCB1* loss; 6 patients were explored by NGS and none of them displayed *SMARCB1* gene mutations [193]. More recently, Jia et al. reported the molecular characterization of 20 RMC patients: all cases displayed protein loss; 55% showed concurrent hemizygous loss and translocation of *SMARCB1*, 30% with homozygous loss of *SMARCB1,* and 15% without structural or copy number alterations of *SMARCB1* despite protein loss; targeted sequencing provided evidence about the existence of a pathogenic somatic mutation in 1 of the 3 cases that were negative by FISH [194]. Tumors pertaining to the three subsets associated with different FISH findings displayed comparable clinicopathologic features; the only peculiarity was related to the cases with homozygous *SMARCB1* deletion being associated with the solid growth pattern, whereas tumor-bearing *SMARCB1* translocations were more associated with reticular/cribiform growth [194].

Hong et al. have developed patient-derived RMC models based on loss-of-function fusion events in one *SMARCB1* allele and loss of the other allele; through functional experiments, it was shown that RMC requires the loss of *SMARC1B* for survival [195]. Using loss-of-function genetic screens and small-molecule screen, it was found that the ubiquitin-proteasome system was essential in RMC: proteasome inhibitors caused G2/M arrest of RMC cells caused by cyclin B1 accumulation and cell apoptosis [195]. These observations support clinical trials based on the use of proteasome inhibitors for the treatment of RMC patients [195].

## 14. Genetic Alterations of Tubulocystic Renal Carcinoma (TCRCC)

In 1997 MacLennan et al. reported the existence of renal cancers that microscopically consisted of well-defined cystic lesions lined by hobnail-shaped cells with low mitotic activity and with a low propensity for recurrence and metastasis [196]. These tumors were classified as low-grade collecting duct carcinoma; immunohistochemical markers suggested a collecting duct origin for these tumors [196]. Subsequent studies have supported the idea that low-grade collecting duct carcinoma and TCRCC are synonymous of the same clinicopathologic entity. The microscopic appearance was characterized by the presence of variable-sized cystically dilated tubules lined by a single layer of epithelium [197]. Immunohistochemistry and ultrastructural analysis supported features of proximal convoluted tubules and distal nephron; gene expression profiling supported a unique molecular signature, different from other RCC types [197].

Recent studies support the existence of TCRCC as a rare peculiar subtype of RCC. In fact, Lawrie et al. performed miRNA expression analysis and targeted next-generation sequencing mutational profiling on 13 cases of TCRCC: the expression profile of some miRs, such as *miR-155* and *miR-34a*, that were downregulated was clearly different from that observed in PRCC; the gene sequencing showed recurrent mutations of *ABL1* and *PDGFRA* genes, both genes being only rarely mutated in other RCC types [198]. More recently, Sarungbam et al. performed a molecular characterization of 10 cases of pure TCRCC by targeted next-generation sequencing and FISH analysis for *X* and *Y* chromosomes: all these carcinomas displayed combined losses at chromosomes *9* and gains at chromosome *17*, and loss of chromosome Y; none of these tumors displayed mutational profiles typical of other RCCs; recurrent mutations in chromatin-modifying genes, *KMT2C* and *KDM5C*, were detected in about 25% of tumors; non *ABL1* and *PDGFRA* mutations were detected [199]. Thus, TCRCC demonstrates genomic features distinct from other subtypes of RCC.

## 15. Wilms Nephroblastoma

Wilms tumor (WT) is largely the most frequent kidney tumor in children (80–90% of the cases). These tumors contain three different histological components: A mesenchymal component resembling primitive fetal mesenchyme; an epithelial component resembling fetal renal tubules and glomeruli; a blastomatous component made by clusters of blast cells that contributed to the definition of these tumors as nephroblastoma. The histopathological features of WTs may be variable and usually the presence of all these histological components is a favorable determinant; unfavorable elements are represented by diffuse anaplasia and the predominance of the blastomatous component.

Initial studies have shown genetic abnormalities of *WT1* gene, Wnt-activating mutations of *CTNNB1* and *WTX*, abnormalities of *11p15* copy number, and methylation [200]. Subsequent genetic studies of large cohorts of WT patients have identified new mutations: recurrent mutations of the miRNA-processing gene *DROSHA* (observed in about 12% of cases) and non-recurrent mutations in other genes of this pathway (*DICER1, DGCR8, XPO5,* and *TARBP2*), associated with the downregulation of miRNA expression in a subset of WTs [201]. Recurrent mutations at the level of the homeodomain of *SIX1* and *SIX2* genes involved in the control of renal development, particularly frequent in WTs with blastemal histology (18% of cases), as well as *DROSHA* mutations (18% of cases) [202]; mutations of *MYCN, SMARCA4,* and *ARID1A* [203].

The most recurrent gene mutations occurring in high-risk subgroups of WT patients subdivided into those exhibiting a favorable histology (FHWT) that subsequently relapsed and those with diffuse anaplasia (DAWT) were defined: recurrent *DROSHA, DGCR8,* and *SIX1/2* homeodomain genes were observed in FHWT [204]; recurrent *TP53* alterations are observed in DAWT, with 48% of cases showing *TP53* mutations, 11% copy loss without mutation: patients with stage III/IV DAWTs had lower relapse and death rates than those with *TP53* abnormalities [205]. Another study showed the frequent occurrence of insertion/deletion *MLTT1* (a gene known to be involved in transcriptional elongation during early development) mutations, associated with altered binding to acetylated histone tails: these tumors show an increase in *MYC* gene expression and *HOX* genes dysregulation [206].

The Children Oncology Group and Target initiative published in 2017 a fundamental study reporting a genome-wide sequencing, mRNA and miRNA expression analyses, DNA copy number, and DNA methylation analyses in 117 WTs, followed by targeted sequencing of 651 WTs [207]. In addition to genes previously found to be mutated in WTs (*WT1, CTNNB1, AMER1, DROSHA, DGCR8, XPO5, DICER1, SIX1, SIX2, MLLT1, MYCN,* and *TP53*), this study discovered as frequently mutated in WTs also *BCOR, BCORL1, NONO, MAX, COL6A3, ASXL1, MAP3K4,* and *ARID1A* genes [207]. *TP53* was the most frequently mutated gene in the discovery set, enriched in DAWT histology; importantly, mutations in *TP53* were significantly associated with DAWT histology (56/118 DAWT and 9/533 FHWT); frequently, *TP53* mutations display a lower allelic fraction, consistent with the role of *TP53* as a secondary mutation in WTs [207]. *CTNNB1* was the most frequently mutated gene with global frequency of 13.5%; *CTNNB1* mutations were much more frequent among FHWT (16%) than among DWAT (1.7%); analysis of co-occurrence mutations showed a significant co-occurrence of *CTNNB1* mutations *WT1* (about 39% of tumors with *CTNNB1* mutations also had *WT1* mutations and about 74% of tumors with *WT1* mutations also had *CTNNB1* mutations) [207]. A significant co-occurrence was also observed between *DROSHA* and *SIX1/SIX2* mutations (15% of tumors with *DROSHA* mutations also had mutations in *SIX1* or *SIX2*, and 23% tumors with *SIX1* or *SIX2* mutations also had *DROSHA* mutations) [207].

At the level of CNAs, WTs are characterized by gains and losses of entire chromosomes or chromosomal arms, such as gains of *1q, 6,* and *12* and loss of *4q, 16q, 17p, 14, 11,* and *22*; gain of *1q* was shown in about 48% of cases; gain of *1q* was not concurrent with any recurrent mutation, suggesting a possible role as a secondary event; amplification of *2p24* including *MYCN* locus, was found in 11.5% of FHWTs and 25.5% DAWTs; loss of *17p* correlated with *TP53* mutations, as well as loss of *4q* and *14q* [207]. Gain of chromosomal segments containing *6q16*, the location of *LIN28B*, was observed in 25.5% of cases and was related to the gain of whole chromosome *6* [207]. Chromosomal loss at *9q22* caused recurrent loss of *MIRLET7A* gene family: *MIRLET7A1* (5%), *MIRLET7A2* (18%), *MIRLET7A3* (/21%) [207]. Gene expression analysis allowed the stratification of FHWT into six clusters: cluster 1 was characterized by *LIN28B* gain, *MIRLET7A* loss, *1q* gain, *WT1* loss, and absence of the most recurrent gene mutations; cluster 2 is characterized by frequent *DROSHA, DGCR8, SIX1,* and *SIX2* mutations, *11p15* methylation; cluster 3 is characterized by frequent *MLLT1, WT1, CTNNB1,* and *WTX* mutations, *WT1* and *WTX* loss, and *MYCN* amplification; cluster 4 is characterized by recurrent *WT1*, *CTNNB1,* and *WTX* mutations and *11p15* methylation; cluster 5 is characterized by the expression of genes involved in oxidative phosphorylation; cluster 6 is characterized by the absence of recurrent mutations, frequent *WT1* loss, and *Let7a* loss [207].

This study showed that WTs: (i) Derive from the cooperation of multiple genetic events; (ii) display different genetic alterations, associated with differential gene expression profiles; (iii) have multiple driver genes, the majority being altered in <5% of tumors; (iv) display mutations at the level of genes with common functions, mainly represented by genes involved in early renal development or epigenetic regulation [207].

Recurrent hot spot mutations have been found in ENL YEATS domain in WTs [208]. ENL protein is a reader of histone acetylation through its YEATS domain. Using human and mouse cellular models, evidence was provided that ENL mutants induce gene expression changes that promote a premalignant condition and in nephrogenesis models induce the formation of undifferentiated cellular structures resembling those observed in WTs [208]. At mechanistic level, these ENL mutations exhibit a function similar to their normal counterpart, occupying similar target genomic loci, but with a clearly increased occupancy, leading to a pronounced increase in the recruitment and activity of transcription elongation machinery, thus enforcing the rate and the level of gene transcription of these target genes [208].

Wilms tumors are characterized by persistent embryonic kidney tissues and arrested cellular differentiation. WTs often evolve from pre-cancerous clonal expansions [209]. To discover potential precursors of WTs, Coorens et al. used somatic mutations to infer the phylogenetic relationship that may occur between kidney tumors and corresponding normal tissues (blood) [209]. To perform this analysis, these investigators initially investigated some children with unilateral WTs and sampled tumor, blood and normal kidney tissue specimens from the same individuals: in two of the three cases analyzed, mosaic mutations in normal kidneys that were present in the corresponding tumor, but absent from blood were observed [209]. Several features of these mutations observed in normal kidney tissue suggest that they can be defined as clonal expansions [209]. Importantly, the study of additional 23 cases of WTs showed evidence of clonal nephrogenesis in 53% of cases with unilateral disease and 100% of those with bilateral disease [209]. These observations suggested that clonal expansions in histologically normal kidney tissue as an atypical outcome of renal tissue development, antedating WT development; a direct phylogenetic link between clonal expansions, H19 hypermethylation, and the formation of cancer, thus supporting the view that these clonal expansions are an epigenetic progenitor of cancer; however, at variance with precursors of adult cancer, clonal nephrogenesis generated histological and functionally normal kidney tissue [209].

## 16. RCCs with Sarcomatoid (sRCC) Features

sRCC is a very aggressive form of RCC, characterized at histological level by the presence of a cellular component that has lost the epithelial features and has acquired mesenchymal features with spindle cells, high cellularity and cellular atypia; sarcomatoid features are observed in 5–10% of CCRCC and CHRCC and in 2–3% of PRCC [210,211,212].

sRCC is not a distinct RCC subtype, but represents a shift in the epithelial differentiation to mesenchymal differentiation in the context of pre-existing RCC; this conclusion is supported by two lines of observations: both an epithelial and a mesenchymal component is present in these tumors; both the epithelial and sarcomatoid components share the large majority of gene mutations, copy number alterations, and X-chromosome inactivation patterns [213]. In spite of these similarities of the epithelial and mesenchymal components suggesting a common origin, several remarkable differences exist between these two components strongly suggesting the evolution of sarcomatoid elements from carcinomatous elements by acquisition of additional genetic abnormalities: (i) Increased burden of cancer driver mutations and CNAs in sarcomatoid elements; (ii) existence of some sarcomatoid-specific mutations, such as *TP53, ARID1A,* and *BAP1* mutations [214] and TGFβ regulator *RELN* and *PTEN* mutations [215]; (iii) several genes involved in epithelial-to-mesenchymal transition display an increased expression in the mesenchymal component compared to the epithelial tumor components [212]; (iv) sarcomatoid components of these tumors displayed increased Aurora kinase-1 expression, supporting a potential role for increased mTOR activation as a driver of mesenchymal shift [216].

Malouf et al. reported the mutational analysis of 26 sRCCs and showed that *TP53* (42%), *VHL* (35%), *CDKN2A* (27%), *NF2* (19%) were the most frequently altered genes [217]. In a more recent report, these authors performed a detailed analysis of targeted sequencing of sRCCs, including also paired sequencing of epithelial and mesenchymal components isolated by microdissection. The most recurrent mutations in these patients involved *VHL* (72%), chromatin remodeling genes *SETD2* (40%), *PBMR1* (34%) and *BAP1* (26%), *TERT* promoter (18%), *PTEN* (14%), *TSC2* (12%), and Hippo pathway members *NF2* (10%) and *FAT1* (10%) [218]. The most altered pathways involved *VHL* (72%), chromatin remodeling genes (72%), MTOR pathway (50%), DNA repair (30%), and the Hippo pathway (20%) [218]. It is of interest to note that concerning the chromatin remodeling genes, in addition to *SETD2, PBRM1,* and *BAP1* mutations, were observed also mutations of *ARID1A* and *ARID1B* genes and of several genes acting as epigenetic regulators [218]. In 23 patients the genomic profiles of paired epithelial and mesenchymal components were compared, showing that: *SETD2* and *TERT* alterations markedly differed between the two components; one tumor harbored *NF2* and *CDKN2A* mutations exclusively in the mesenchymal component; two tumors harbored *TP53* mutations exclusively in the mesenchymal component [218]. Hippo pathway alterations were clearly more frequent in sRCC compared to non-sRCC [218]. Hippo-mutant sRCCs showed YAP/TAZ upregulation, thus showing that Hippo pathway is activated in these tumors; furthermore, Hippo pathway inhibition or restoration of normal NF2 expression inhibited the proliferation and invasiveness of sRCC [218].

Ito and coworkers reported a detailed analysis on CNAs occurring in 17 sRCCs, showing that these tumors are associated with a high rate of chromosomal abnormalities involving losses of *9q, 15q, 18p/q,* and *22q* and gains of *1q* and *8q* occurring at significantly higher frequencies compared to the corresponding non-sarcomatoid RCCs [219]. Among sRCC patients, those with >9 chromosomal abnormalities showed significantly worse overall survival than those with <9 copy number alterations [219].

In addition to sRCCs that are among the most aggressive RCCs, a high proportion of aggressive RCCs is observed at the level of the group of RCCs with unclassified histology (uRCC); these tumors are poorly characterized at molecular level. Chen et al. [64] reported the extensive molecular characterization of 62 primary high-grade uRCCs: sequencing analysis showed recurrent mutations at the level of 29 genes, the most frequent being *NF2* (18%), *SETD2* (18%), *BAP1* (13%), *KMT2C* (10%), *MTOR* (8%), *PTEN* (7%), and *TSC1* (7%); integrated molecular analyses showed the existence of a subset (26% of uRCCs) characterized by *NF2* loss, dysregulated Hippo-YAP pathway and poor survival and of another subset (21% of uRCCs), characterized by recurrent mutations of *MTOR*, *TSC1, TSC2,* or *PTEN*, hyperactive MT OR signaling and a better clinical outcome [64]. The frequent *NF2* abnormalities and the consequent dysregulation of the Hippo pathway represent a common feature of both sRCC and uRCC and support the targeting of this pathway for the therapy of a subset of these aggressive RCCs [220].

sRCCs are often metastatic and show a poor response to current therapeutic approaches. However, recent studies suggest that these tumors could be sensitive to immunotherapy treatments based on immune check inhibitors. Several studies have shown that PD-L1 expression is increased in sRCCs: importantly, PD-L1 expression is increased at the level of the sarcomatoid and not at the level of the epithelial component of these tumors [221,222]. Data from tumors of patients enrolled in clinical trials involving treatment with immune check inhibitors confirmed high levels of PD-L1 expression in sRCC of clear-cell type, with ≥50% of patients exhibiting a PD-L1 expression ≥1% of tumor [223] or microenvironment immune-infiltrating cells [224]. In a part of these patients elevated PD-L1 expression seems to be related to a molecular mechanism dependent upon *9p24.1* amplifications [225]. Ongoing clinical trials support the immunogenic potential of sRCCs both at the level of gene expression profile and at the level of response to treatment with immune check inhibitors combined with VEGF inhibitors [226].

## 17. Conclusions

RCC is among the top ten most commonly diagnosed cancers worldwide, accounting for 5% and 3% of all adult malignancies in men and women, respectively and representing the 7th most common cancer in men and the 10th most common cancer in women. Approximately, 2–3% of all RCCs are hereditary and several autosomal dominant syndromes have been identified, each with a distinct genetic basis and phenotype, the most common one being VHL disease. CCRCC is the most frequent RCC, accounting for about 70–75% of all cases and for the majority of renal cancer-caused deaths, followed by PRCC and CHRCC. Site of origin within the nephron is a major determinant in this classification in three major subtypes.

These various types of RCC have been defined on the basis of their histological appearance, the presence of distinct driver mutations, varying clinical course, and different responses to therapy. Extensive genomic, epigenomic, and transcriptomic profiling studies support that the different types of RCC are different diseases each different from the other. Integrated, multi-platform analysis of RCCs showed that these tumors can be subdivided into nine molecular-based RCC subtypes: (i) Three different subtypes were predominantly CCRCC cases and were designated CC-e.1, CCe.2, and CC-e.3, characterized by individual molecular features and by intermediate, better, and worse prognosis, respectively; (ii) four different subtypes of predominantly PRCC cases, P-e.1a, P-e1.b, P-e.1.2, and P-CIMP-e; (iii) one subtype of predominantly CHRCC [227]. These different subtypes can be further subdivided according to differences in patient survival or at the level of alterations of specific biochemical pathways, such as hypoxia, metabolism, Hippo pathway, MAP kinase, PI3K-AKT, NRF2-ARE, mTOR, and immune checkpoint [227].

These studies have allowed fundamental progresses in our understanding of the molecular mechanisms involving RCC development. Thus, molecular studies in CCRCC have defined the dysregulation of the *VHL* gene as an almost universal initial, founding event, followed by different types of additional genetic events involving *PBRM1, KDM5C, SETD2,* or *BAP1* that differentially dictate disease progression and aggressiveness [178,179]. CCRCC tumors with *PBRM1* mutations respond to targeted therapy differently than tumors with *BAP1* mutations [228]. These studies have strongly supported the utility of molecular studies, in addition to histological studies, to stratify CCRCC patients and to identify new potential therapeutic targets.

CCRCC is the prototype of a cancer resistant to conventional chemotherapy and radiotherapy and there is consistent hope that a better understanding of the molecular pathogenesis of RCC could contribute to the definition of more efficacious treatments. The discovery of abnormalities of several pathways has led to the approval of six different types of drugs for the treatment of metastatic RCC: inhibitors of VEGFR, mTORC1, c-MET, and FGFR; cytokines; anti-PD1/PDL1 immune checkpoint inhibitors [229]. These treatments have led to an improvement of metastatic RCC patients; however, in most of cases, the responses to these agents have been limited [229].

In the past two decades there has been a consistent improvement in the number of RCC therapies, characterized by a first period related to the development of targeted approaches based on the identification of targetable altered pathways, followed by a second period related to the development of immune-oncological therapies based on the stimulation of host immune system to promote an efficient immunological anti-tumor response; finally, the ongoing third period based on combination therapies that could improve survival in metastatic RCC [230].

RCC patients with localized stage I to III disease are treated with surgical resection; about one-third of these patients eventually recur; furthermore, 15% of RCC patients present with locally advanced or metastatic RCC, for which surgery is a noncurative treatment. For this last type of patients, over the past decade the standard of care has undergone significant changes and is currently in a state of continuous revisions. Anti-angiogenic inhibitors were the first targeted therapies approved for RCC treatment. The rationale for their use was related to the very frequent VHL alterations observed in RCCs and responsible for activation of hypoxia signaling pathway in these tumors [230]. Thus, sorafenib was approved by FDA in 2005 and was followed by other VEGFR small molecular TKIs, such as pazopanib and axitinib [230]. Early studies have suggested a sensitivity of a small subset of RCC patients to immunotherapy-based approaches using IL2 or IFN-α. The development of a more modern era of anticancer immunotherapy was based on the use of anti-CTLA4 and anti-PD-1/PD-L1 checkpoint inhibitors. These inhibitors have the capacity to block the inhibitory effects on the immune anticancer response existing in various tumors, including RCC. Many trials have examined the effect of immunotherapy alone or in combination with antiangiogenic TKIs and were shown to be superior to the existing standard of care [230].

Currently, sunitinib, pazopanib, nivolumab plus ipilimumab, pembrolizumab plus axitinib, avelumab plus axitinib are considered first-line treatments. Two recent clinical trials based on combination therapy strongly support the great potentialities of this approach to improve the survival of mRCC patients. Thus, the KEYNOTE-426 trial evaluated in first-line mRCC patients the safety and efficacy of pembrolizumab (anti-PD1) plus axitinib (VEGF inhibitor) versus sunitinib: patients treated with pembrolizumab plus axitinb had increased 12-month overall survival at 90%, compared to sunitinib at 78%; at 15.1 months, the progression-free survival was longer for pembrlizumab plus axitinib compared to sunitinib; the rate of adverse events was slightly higher in the pembroliuzumab plus axitinib arm than in the sunitinib arm [231]. The overall survival, progression-free survival, and overall response rate were not significantly influenced by tumor PD-L1 expression and by patient risk stratification [231]. The updated results of this trial were presented at the last ASCO Meeting, showing that 74% of the patients were alive from the pembrobilumab plus axitiinb arm at 24 months, compared to 66% in the sunitinib arm; the median overall survival was 35.7 months for patients treated with sunitinib and not reached for those treated with pembrolizumab plus axitinib; the progression-free survival was 15.4 months versus 11.1 months; the overall response rate was 60.2% with pembrolizumab plus axitinib and 40% with sunitinib; the median duration of response was 23.5 months with pembrolizumab plus axitinib versus 15.9 months with sunitinib [232]. The analysis of treated patients stratified according to the tumor risk category showed that the benefit in terms of overall survival, progression-free survival, and overall response rate related to pembrolizumab plus axitinib therapy was limited to intermediate- and high-risk mRCC patients [232].

Another combination therapy study was the JAVELIN Renal 101 trial comparing axitinib plus avelumab, an anti-PDL1 antibody, with sunitinib in first-line metastatic RCCs: the treatment with axitinib plus avelumab increased the median PFS compared to subitinib (13.8 months vs. 8.4 months); tumor PD-L1 positivity did not modify progression-free survival or the overall response rate; the safety profile was comparable in the two arms of treatment [233]. A recent update of this study confirmed the improvement of progression-free survival in the axitinib plus avelumab arm compared to sunitinib arm; overall survival data were still immature for evaluation [234].

Very recent studies further supported the rationale to therapeutically target pathways altered in RCCs. Thus, a very recent study presented at the last Genitourinary Cancer Symposium reported promising results of a phase I/II study involving the study of MK-6482, a HIF2-α inhibitor [235]. The very frequent VHL loss in CCRCC determines HIF accumulation and activation, and through this mechanism, stimulates blood vessels formation in RCCs. This study involved 55 patients with advanced RCCs who had an average of 3 prior lines of therapies; after a median follow-up of 13 months, the overall response rate was 24%; 74.5% of patients had stable disease, with a disease control rate of 80%; median PFS for whole population was 11.0 months; for favorable, intermediate, and poor risk RCC patients the PFS was 16.5, 11, and 6.9 months, respectively [235].

A report by Jonesch et al. showed the preliminary results of a phase II study (NCT 03401788) involving the treatment of 61 patients with germline VHL mutant, localized/nonmetastatic CCRCC, common lesions outside the kidney (non-RCC tumors such as hemangioblastomas (80%) and pancreatic lesions (50%)); about 28% of the patients displayed objective responses and about 87% of patients showed decrease in the size of target lesions [236].

The current, updated ESMO guidelines for treatment of advanced/metastatic RCC indicate that: the combination of pembrolizumab and axitinib should be considered as a front-line therapeutic option for patients with advanced disease, irrespective of prognostic groups and of the PD-L1 biomarker status; the combination nivolumab and ipilimumab should be considered in patients with intermediate/poor risk status; VEGF-targeted therapy is recommended for those patients where pembrolizumab/axitinib or nivolumab/ipilimumab are not available or contraindicated.

Given the heterogeneity of RCCs and the variability of their response to immunotherapy-based combination treatments, it will be of fundamental importance to acquire a better understanding of the genetic and epigenetic features of RCC patients who respond to these treatments.

## Figures and Tables

**Table 1:** Hereditary RCC syndromes, associated molecular alterations, and clinical manifestations.

Syndrome	Gene (chromosome)	Protein	Clinical Manifestations	Histology
Von Hippel-Lindau Syndrome	VHL (3p25)	pVHL	CCRCC, Pheochromocytoma, pancreatic endocrine tumors, CNS, and retinal hemangioblastomas	CCRCC Clear cell papillary
Hereditary Papillary RCC (HPRCC)	MET (7q31)	MET	Type 1 papillary RCC	Papillary type 1
Cowden Syndrome	PTEN (10q23.31)	Phosphatase and tensin homolog	Dermatological lesions. breast cancer, thyroid cancer, endometrial cancer	Papillary Chromophobe CCRCC
BAP1 Hereditary Syndrome	BAP1 (3p21)	BRCA1-associated protein-1	Uveal and cutaneous, melanoma, malignant mesothelioma, and/or lung adenocarcinoma	Undefined
Hereditary paraganglioma-pheochromocytoma syndromes	SDHA (5p15.33) SDHB (1p36.1-p35) SDHC (1q23.3) SDHD (11q23,.1)	Succinate dehydrogenase	Bilateral and extra-adrenal pheochromocytoma, paraganglioma, RCC, and other malignancies	SDH-deficient RCC (solid nests or tubular architecture with variable cysts; vacuolated cells with eosinophilic cytoplasm)
Hereditary leiomyomatosis and renal cell carcinoma (HLRCC)	FH (1q42,.1)	Fumarate hydratase	RCC, leiomyomas of skin and uterus (leiomyosarcoma), malignant pheochromocytoma/paraganglioma	HLRCC-associated RCC papillary type 2
Birt-Hogg-Dubé (BHD) Syndrome	FLCN (17p11.2)	Folliculin	RCC (hybrid oncocytic and other types), fibrofolliculomas, pulmonary cysts	Chromophobe Oncocytoma Hybrid CCRCC
MITF-associated susceptibility to melanoma and RCC syndrome	MITF (3p14.1)	Microphtalmia- associated transcription factor	Melanoma, pancreatic cancer, and/or pheochromocytoma	Undefined

**Table 2:** Molecular abnormalities of main sporadic renal cell cancers (RCCs).

RCC Subtype	Somatic Mutations or Alterations	Copy Number Variations or Translocations	Prognostic Implications of Genomic Alterations
**CCRCC**	Mutations in *VHL, PBMR1, SETD2, BAP1, KDM5C, TERT promoter, MTOR*	Loss of chromosomes *3p, 14q, 9p, 6q, 8p,15q* Gain of chromosome *5q*	*VHL*: no association *PBMR1*: greater survival/no benefit *BAP1, SETD2, CDKN2A, TP53*: reduced survival *PDH* genes, Ribose sugar metabolism genes: reduced survival
**PRCC, type I**	Mutations in *MET, NRF2, CUL3*	Gains of chromosomes 3, 7, 16, 17	*CDKN2A, PBMR1, TP53*: reduced survival *DKK1/SFRP1:* unmethylation: reduced survival
**PRCC, type II**	Mutations in *CDKN2A, CDKN2B, TERT, NF2, FH, MET, SETD2*	Gains of chromosomes 7, 16, 17, 5q Loss of chromosomes: 3p, 14q, 22q Translocation of *TFE3*	*CDKN2A, TP53*: reduced survival *DKK1/SFRP1:* unmethylation: reduced survival
**CHRCC**	Mutations in *TP53, PTEN*	Loss of chromosomes 1, 2, 6, 10, 13, 17, 21	*PTEN, CDKN2A*: reduced survival *DKK1/SFRP1:* unmethylation: reduced survival Metabolically divergent tumors: highly reduced survival
**RMC**	Mutations in *SMARCB1*	Amplification of *ABL*	Unknown
**TCRCC**	Mutations in *ABL1, PDGFRA*	Gains of chromosomes: 7,17	Unknown
**Wilms Nephroblastoma**	Mutations in *TP53, AMER1, CTNNB1, WT1, DROSHA, DGGR8, DICER1, SIX1/SIX2, SMARCA-4, MLTT1*	Loss of chromosomes 1p, 16q, 1q, 17p, 4q, 14q, 11q, 11p15.	*TP53, SIX1/SIX2, DROSHA/DGGR8*: reduced survival Loss of chromosomes *1p, 1q, 11p15,* and *16q*: reduced survival

**Table 3:** Evolutionary patterns of clear-cell RCC (CCRCCs) that are associated with the development of different metastatic potentials. Tumors that follow a linear pattern of evolution have a limited intratumor heterogeneity (ITH), a low genomic instability index (GII), few mutations in addition to VHL and a low fraction of their genome affected by copy number alterations (SCNAs), and display a low metastatic potential. The branched pattern of tumor progression implies high ITH and GII, the progressive acquisition of additional mutations after *VHL* loss, with early acquisition of *PBMR1* mutations and then subclonal acquisition of additional genetic alterations (*SETD2* mutations, PI3K-AKT-mTOR pathway mutations,), associated with a slow metastatic development. The punctuated pattern is characterized by high GII and low ITH, early chromosome *9p* and *14q* loss, acquisition of multiple driver mutations, including *BAP1* mutations and rapid acquisition of a metastatic potential.

Evolution Pattern	Early Events	Primary Tumor	Genomic Characterization	Metastatic Potential
Linear	Chr 3p loss VHL inactivation Initial clonal expansion	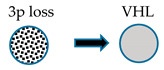	Low GII Low ITH	Non-Metastatic
Branched	Chr 3p loss VHL inactivation Initial clonal expansion	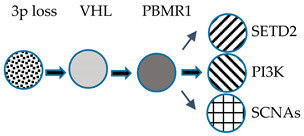	High GII High GII	Slow Progression
Punctuated	Chr 3p loss VHL inactivation Initial clonal expansion	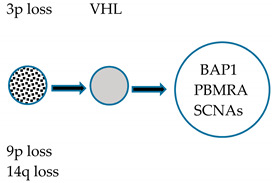	High GII Low ITH	Rapid Progression
